# Stochastic modelling of a three-dimensional glycogen granule synthesis and impact of the branching enzyme

**DOI:** 10.1371/journal.pcbi.1010694

**Published:** 2023-05-19

**Authors:** Yvan Rousset, Oliver Ebenhöh, Adélaïde Raguin

**Affiliations:** 1 Institute for Quantitative and Theoretical Biology, Heinrich-Heine University, Düsseldorf, Germany; 2 Institute for Computational Cell Biology, Heinrich-Heine University, Düsseldorf, Germany; 3 Cluster of Excellence on Plant Sciences (CEPLAS), Heinrich-Heine University, Düsseldorf, Germany; University of California Riverside, UNITED STATES

## Abstract

In humans, glycogen storage diseases result from metabolic inborn errors, and can lead to severe phenotypes and lethal conditions. Besides these rare diseases, glycogen is also associated to widely spread societal burdens such as diabetes. Glycogen is a branched glucose polymer synthesised and degraded by a complex set of enzymes. Over the past 50 years, the structure of glycogen has been intensively investigated. Yet, the interplay between the detailed three-dimensional glycogen structure and the related enzyme activity is only partially characterised and still to be fully understood. In this article, we develop a stochastic coarse-grained and spatially resolved model of branched polymer biosynthesis following a Gillespie algorithm. Our study largely focusses on the role of the branching enzyme, and first investigates the properties of the model with generic parameter values, before comparing it to *in vivo* experimental data in mice. It arises that the ratio of glycogen synthase over branching enzyme reaction rates drastically impacts the structure of the granule. We deeply investigate the mechanism of branching and parametrise it using distinct lengths. Not only do we consider various possible sets of values for these lengths, but also distinct rules to apply them. We show how combining various values for these lengths finely tunes glycogen macromolecular structure. Comparing the model with experimental data confirms that we can accurately reproduce glycogen chain length distributions in wild type mice. Additional granule properties obtained for this fit are also in good agreement with typically reported values in the experimental literature. Nonetheless, we find that the mechanism of branching must be more flexible than usually reported. Overall, our model provides a theoretical basis to quantify the effect that single enzymatic parameters, in particular of the branching enzyme, have on the chain length distribution. Our generic model and methods can be applied to any glycogen data set, and could in particular contribute to characterise the mechanisms responsible for glycogen storage disorders.

## Author summary

Glycogen is a granule-like macromolecule made of 10,000 to 50,000 glucose units arranged in linear and branched chains. It serves as energy storage in many species, including humans. Depending on physiological conditions (hormone concentrations, glucose level, etc.) glycogen granules are either synthesised or degraded. Certain metabolic disorders are associated to abnormal glycogen structures, and structural properties of glycogen might impact the dynamics of glucose release and storage. To capture the complex interplay between this dynamics and glycogen structural properties, we propose a computational model relying on the random nature of biochemical reactions. The granule is represented in three dimensions and resolved at the glucose scale. Granules are produced under the action of a complex set of enzymes, and we mostly focus on those responsible for the formation of new branches. Specifically, we study the impact of their molecular action on the granule structure. With this model, we are able to reproduce structural properties observed under certain *in vivo* conditions. Our biophysical and computational approach complements experimental studies and may contribute to characterise processes responsible for glycogen related disorders.

## 1 Introduction

Management of energy resources is crucial for an organism to survive, since nutrient availability may vary considerably with time. Moreover, organisms face numerous other stresses that may temporarily increase energy demand. To react to such changes in energy supply and demand, it is apparent that some internal stores are necessary. These stores are filled when nutrients are abundant and depleted when demand exceeds available supply. As direct substrate for catabolic pathways, glucose plays a central role in energy metabolism in most organisms [1]. While plants have evolved to store glucose as starch, animals, fungi, and most bacteria store glucose as glycogen. Both starch and glycogen are branched polymers consisting of glucose monomers, linked into linear chains by *α*-1,4 bonds, and branching points by *α*-1,6 bonds. However, these two macromolecules exhibit rather different physico-chemical properties. In contrast to glycogen, starch is insoluble in water under physiological conditions, and contains high density crystalline regions. These different properties are reflected by distinct branching patterns and chain length distributions (CLD). Functionally, starch and glycogen can be compared to capacitors in electric circuits. The latter are able to store and release electrons depending on current and voltage. Thus, they can be used to stabilise a fluctuating electric signal. Analogously, glycogen and starch can be seen as two different capacitors that both contribute to glucose homeostasis by managing energy resources through time.

While the fine structure of starch has been widely investigated over the past 50 years, less is known on that of glycogen [2]. The characterisation of the detailed structure of glycogen, as well as the interplay between its structural properties and the dynamics of glycogenesis (synthesis) and glycogenolysis (degradation) is unclear. Yet, both glycogen structure and dynamics are of utmost interest for understanding glycogen metabolism and the impact of related genetic variances. For human health, this is particularly important considering the increasing prevalence of glycogen storage diseases (GSDs), as well as diabetes, and other glycogen related disorders.

**Fig 1:**
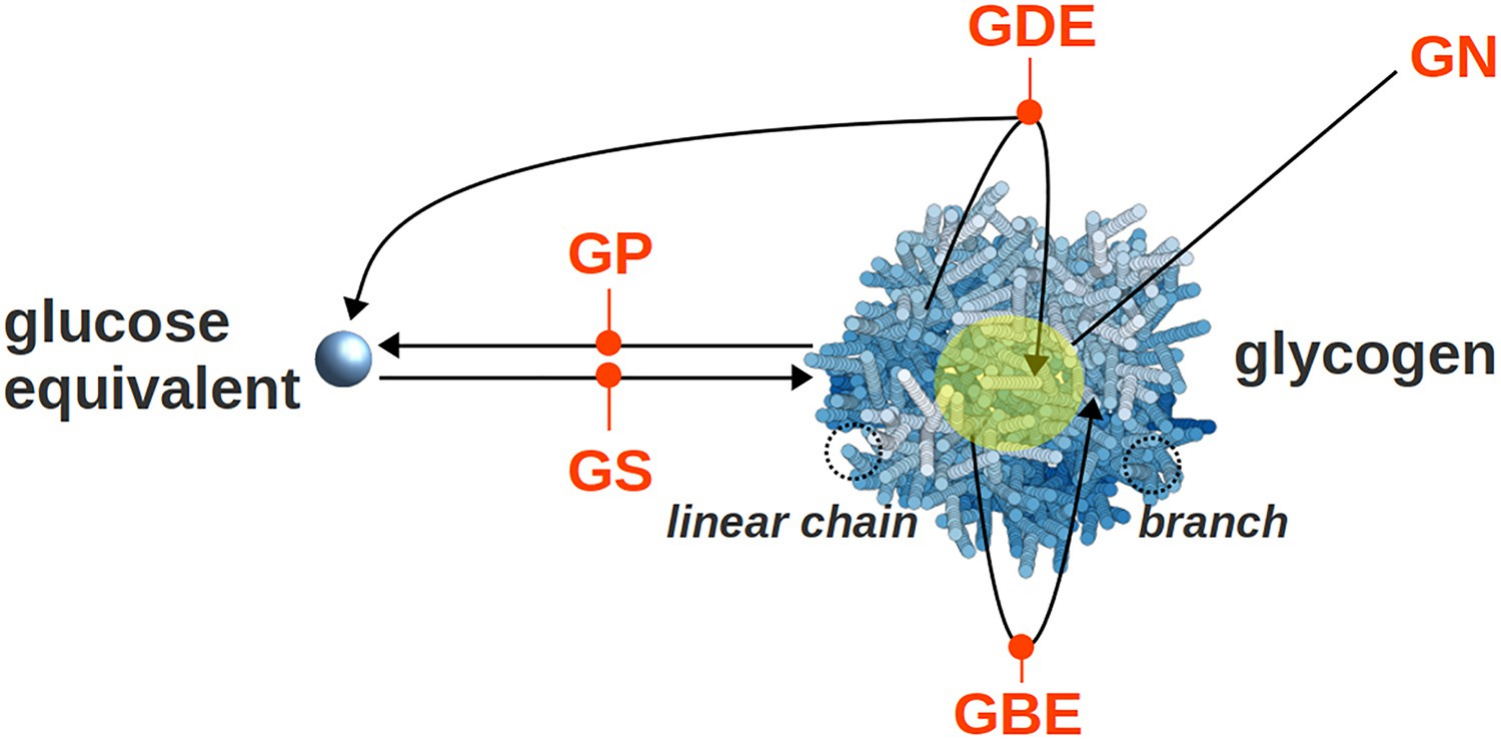
Main enzyme reactions involved in the synthesis and breakdown of glycogen. *In vivo*, the GS and GBE enzymes synthesise glycogen, while the GP and GDE degrade it. Besides, GN is the initial precursor of the granule and stands in its core. Enzymes are noted in orange, glucose residues are in blue, and GN is highlighted with a yellow sphere.

So far, different structures of glycogen have been proposed [3–6], but a structural model that emerges from the detailed underlying enzymatic mechanisms of synthesis and degradation is still lacking. Understanding and precisely describing the biochemistry of glycogen is challenging. With a molecular weight of 10^6^ to 10^7^ g⋅mol^−1^ [7–9], glycogen is a large molecule, even compared to the enzymes involved in its dynamics [10–12]. It is thus expected that, enzymes synthesising, degrading, or otherwise altering glycogen, can only access certain branches near its surface, while many glucose residues near the centre of the molecule remain ‘hidden’. At the macroscopic scale, the structure of glycogen is well known. Drochmans [13] observed two populations of glycogen granules in rat liver. The so-called *α* granules are aggregates of the smaller *β* granules [14–16]. The latter have a radius in the range from 10 to 20 nm, while the radius of *α* granules ranges between 40 and 300 nm [13]. Here, we focus on *β* granules, whose synthesis and degradation both involve a relatively small number of enzymes (see Fig 1). For a comprehensive review, see [17].

To initiate a new glycogen molecule, a chain of 5 glucose units is synthesised and bound to a glycogenin (GN) protein, which forms the core of the final granule [18–21]. Once initiation is completed, the granule is expanded by the two enzymes Glycogen Synthase (GS) and Glycogen Branching Enzyme (GBE). GS is an elongating enzyme that adds one glucose residue to the non-reducing end of an *α*-1,4 linear chain, thereby forming a new *α*-1,4 glucosydic bond. GBE cleaves a small part of a linear chain and creates a new branch by forming an *α*-1,6 glucosydic bond. We call “daughter” the newly formed chain and “mother” the one it is branching from.

Besides synthesis, granules are subject to degradation, that is performed by two other enzymes. Glycogen Phosphorylase (GP) and Glycogen Debranching Enzyme (GDE) respectively shorten and debranch glycogen branches.

Depending on the relative kinetics of these four enzymes (GS, GBE, GP, and GDE) the overall size of the glycogen granule can either increase or decrease. In this article, we choose to focus on glycogen synthesis, and more specifically on the role of the branching enzyme GBE.

Experimental observations [22, 23] of glycogen show an average chain length (CL) around 13 glucose units, which depends on the organism type. A typical peak is observed at low degree of polymerisation (DP), around DP 8, and almost no chains are detected above DP 40 [22–25]. The degree of branching is defined in two ways in the literature. It is most commonly defined as the ratio of *α*–1,6 to *α*–1,4 linkages, but sometimes also as the average number of *α*–1,6 bonds per chain [6]. We will apply the first definition throughout the paper. This ratio is in the range 0.02 − 0.05 in amylopectin (the prime constituent of starch) [26–28] and 0.06 − 0.10 in glycogen [9, 29]. In 1956, Peat et al. [30] introduced the concept of A and B chains. An A chain does not carry any branch, while a B chain does. The A:B ratio is an important characteristics of glycogen granules and an indicator of the branching pattern. Early studies reported an A:B ratio of 1 in glycogen [22, 31], while it is usually greater than 1 in amylose, and ranging from 1.5 to 2.6 in amylopectin [31, 32].

As early as in the 1940s, various hypotheses have been formulated aiming at explaining macroscopic features of glycogen granules. One of them has become known as the Whelan model, which assumes that every chain carries on average two branches. Based on the Whelan model, the nowadays widespread idea emerged that glycogen can be described as a fractal molecule [6, 33, 34]. A fractal glycogen structure is indeed attractive because it can reproduce various structural properties of glycogen. Moreover, it provides a mechanism explaining why glycogen granules seem to have a maximum size. With this model, glycogen becomes too dense at the surface due to an exponential increase of the number of non-reducing ends with the distance from the centre. Thus, steric hindrance prevents synthesis to continue. However, Manners [2] stressed in 1990 that there is no clear evidence that supports a regular branching and therefore a fractal pattern. More recently, further arguments and results against a fractal structure have been raised [35–37]. Besides, independently from any fractal structure considerations, Zhang et al. [38] proposed a mathematical model based on a Monte Carlo approach to numerically simulate glycogen biosynthesis, aiming to support the common idea that steric hindrance limits granule growth. In this model, glucose units are placed on a three-dimensional grid, and the granule biosynthesis is simulated by adding glucose units on any of the 26 neighbouring positions around the end of the growing chain. As a result, limited growth is an emergent property of this model.

Here, we propose a mechanistic model for glycogen synthesis, focussing on the impact of the branching enzyme on the granule structure. We aim at explaining macroscopic and experimentally observable quantities, such as chain length distributions (CLDs), from the underlying enzymatic mechanisms. Such CLDs can be predicted theoretically using numerical inversion of Laplace transforms [39] or kinetic equations [40–42]. However, besides the fact that CLDs are only one of the many quantities that can be measured in the glycogen structures we simulate, our model accounts for complex features for which an analytic treatment is no longer feasible. For instance, these are the complete connectivity of the structure, complex enzyme mechanisms, the helical structure of the linear chains, and steric-hindrance effects. Also, in contrast to the model proposed by Zhang et al. [38], we do not restrict the location of glucose units to a grid, and instead reflect the helical structure of glucose chains, using parameter values derived from biophysical properties. Therefore, our model provides a more flexible and more realistic representation of the three-dimensional granule structure. Specifically, it is designed to study the effect of the enzymatic activity on the structure, and infer model’s parameter values using comparisons to quantitative experimental data. Differently, Zhang et al. [38] focus on explaining the limited growth of glycogen granules. To our knowledge, these scientific questions have so far not been addressed by computational approaches. In this article, we first detail the geometrical and structural features, and enzymatic reactions, taken into consideration in our model. Then, we analyse distinct properties of the model with a specific focus on the effect of the branching enzyme. Finally, we compare the model to experimental data and discuss the parameter values resulting in a best fit, in relation to typical values reported in the experimental literature. In addition, several complementary results justifying our modelling choices are reported in the extensive Supporting information (S1 Text–S5 Text).

## 2 Results

### 2.1 The model

#### 2.1.1 Glycogen structure and geometry

**Fig 2:**
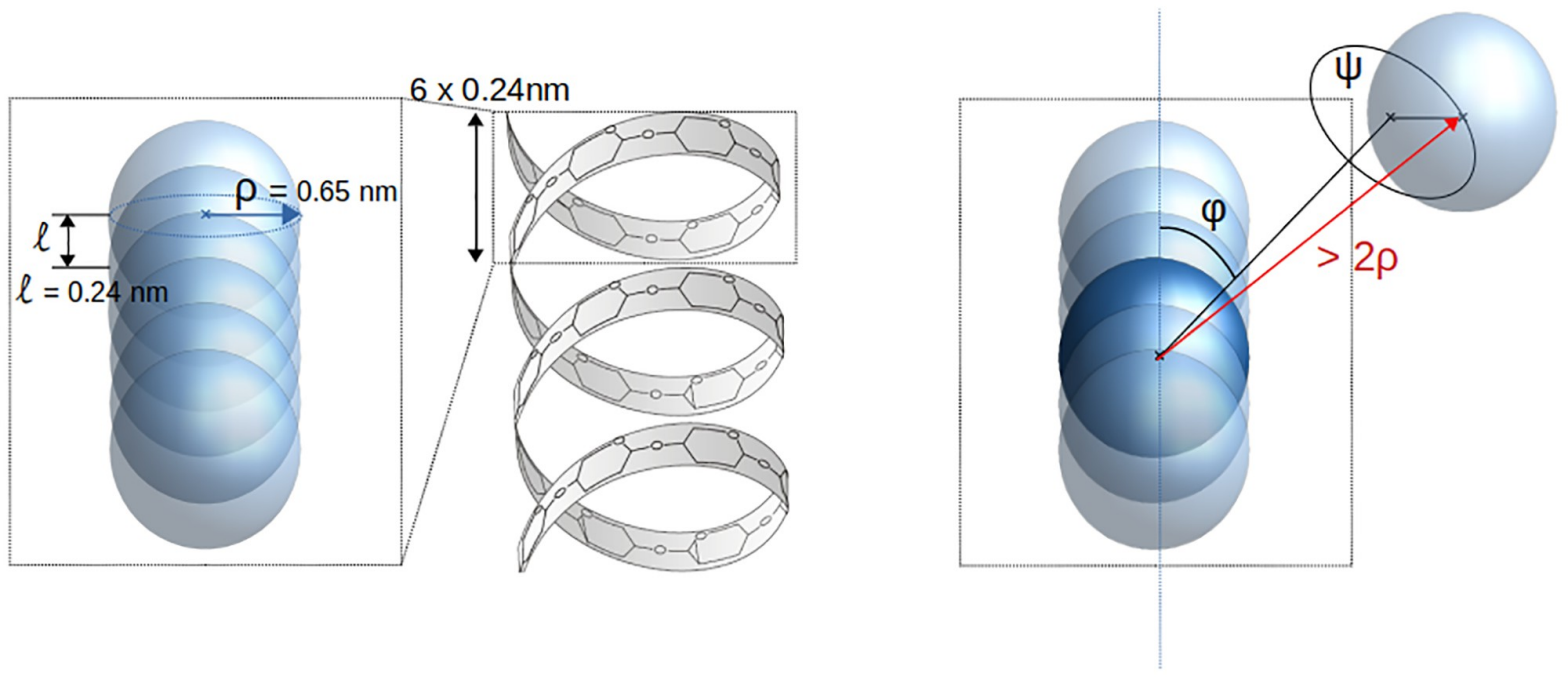
Geometrical description of glucose chains. **Left: Coarse-grained linear chain.** Assuming helical chains, glucose units are described as interpenetrated spheres with radius *ρ* = 0.65 nm. Two consecutive glucoses are distant by *l* = 0.24 nm, which is the radial contribution to the chain length of one glucose in a helical structure. **Right: Description of a branching point.** We generate the direction of the new branch by randomly picking two angles *φ* and *ψ*. The first monomer of the new branch will be located at a distance greater than 2*ρ* to insure no overlapping between the mother and the daughter branches.

We represent glycogen granules as simplified three-dimensional structures, in which every glucose monomer is characterised by its position in space (see Fig 2). To describe the branched tree-like structure, we generalise the simple representation of linear self-avoiding polymers. Using X-ray experiments, Goldsmith et al. [33] characterised in detail how glucose molecules are arranged into helical *α*-1,4 linear chains. The cross-section of the helix has been calculated to be 1.3 nm^2^ with 6 to 7 residues per turn of length 1.4 nm. The radius of the circular cross-section is thus *ρ* = 0.65 nm, and each glucose residue contributes to the chain length by *l* = 0.24 nm. Inspired by these findings, we propose that monomers are described as overlapping spheres with radius *ρ* = 0.65 nm, equal to that of the helix. The validity of this assumption and its impact on our results are presented in detail in Fig A in S1 Text. Besides, the center of consecutive monomers are distant by *l* = 0.24 nm, which corresponds to the contribution of one glucose unit to the chain length, but also involves that the coarse-grained monomer spheres overlap. However, to realistically reflect self-avoidance, two different chains cannot overlap. With these spatial considerations, we ensure that the contribution to the volume by one glucose unit in the model is similar to that of the real helical chain. This way, we provide a description which is simple enough to be easily represented in a computer model, but still realistic enough to reflect the spatial properties of linear chains arranged into helices.

Describing branches formed by *α*-1,6 linkages requires additional geometrical considerations. As illustrated in Fig 2 (Right panel), a branch point is defined by the monomer on the mother chain to which the daughter chain is attached, and two angles defining the direction of the latter. Besides, the anchoring monomer on the mother chain and the first one of the daughter chain are distant by more than 2*ρ*, ensuring that they do not overlap.

Glycogen synthesis is initiated by GN that is located in the centre of the molecule [43]. GN contributes to the total volume of glycogen, and thus we also consider its self-exclusion with any glucose residue of the granule. For the sake of simplicity, we assume that it is a sphere of density 1.3 g ⋅ cm^−3^ [44], which is the typical density of a protein. Accounting for its two sub-units of 38 kDa each [11], we approximate its radius by *ρ*_GN_ ≈ 2.85 nm. Together with GS, which is responsible for elongation, GN may initiate more than a single primary chain, possibly either 2 or 4 [21]. We model this initial core structure with two primary chains pointing out of the GN sphere in opposite directions.

#### 2.1.2 Enzymatic reactions

**Fig 3:**
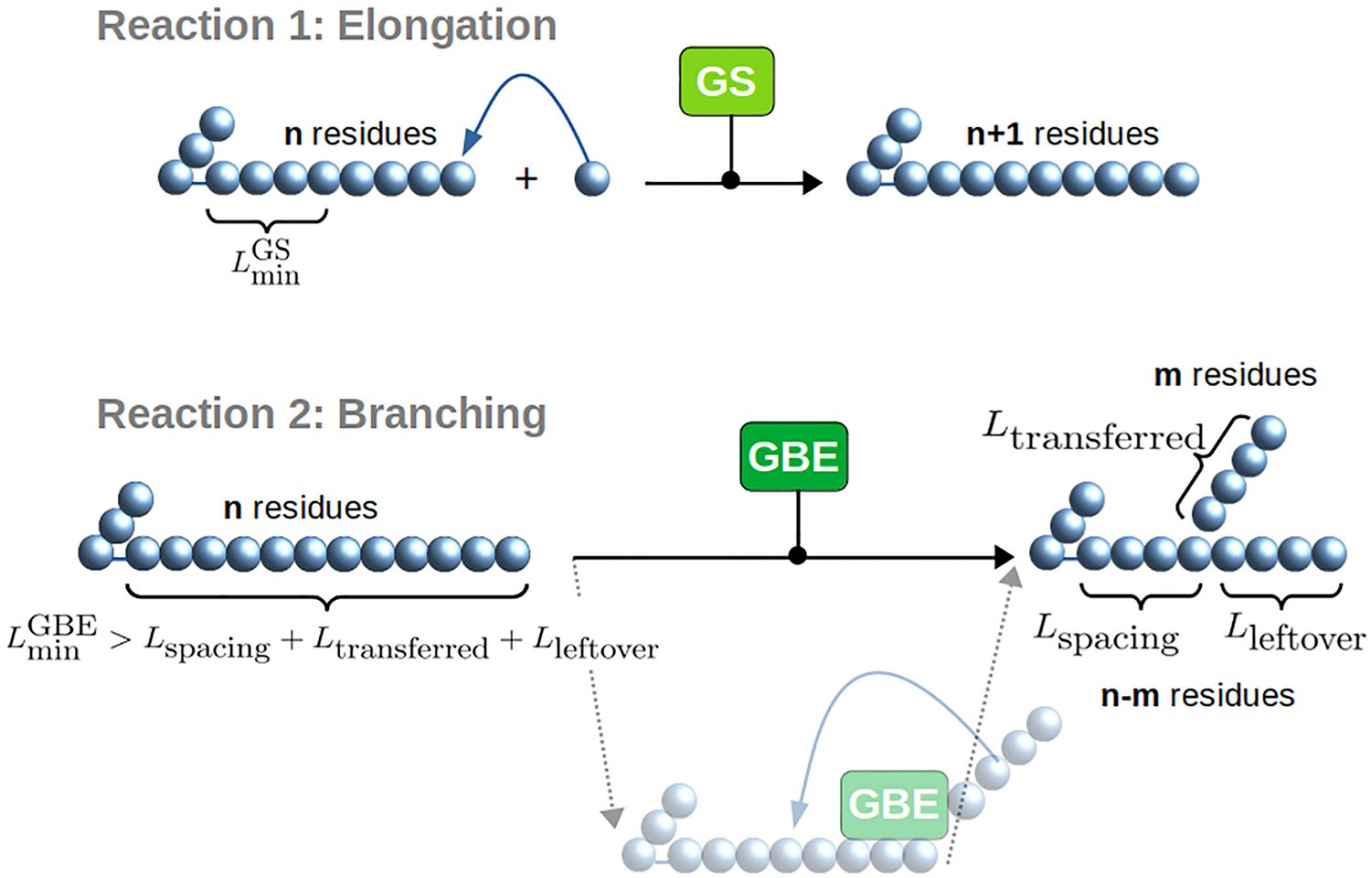
Glycogen synthesis reactions. Glycogen Synthase (GS) catalyses the elongation reaction. It needs a branch with a minimal DP as a substrate and a glucose unit to react. Glycogen Branching Enzyme (GBE) catalyses the branching reaction if the substrate’s DP is greater than the sum of 3 different minimal lengths.

Two enzymes (GS and GBE) are directly involved in glycogen synthesis. Their role is illustrated in Fig 3.

GS binds the non-reducing end of an *α*-1,4 linear chain and elongates it by adding one glucose residue. It is commonly assumed that GS needs a glucose chain primer as a glucose acceptor [45]. In the model, we call $L_{min}^{\text{GS}}$ the minimal chain length of this required primer by GS. As elongation takes place, the chain becomes long enough to be cleaved and branched. This reaction is catalysed by GBE. Just like GS, the action of GBE is also characterised by specific substrate and product lengths as shown in Fig 3. Since less is known for GBE, we tested two models for its mechanism, namely the strict location model and the flexible location model. These are detailed in Fig A and Fig B in S2 Text. While comparing the two models to experimental data, we observed that the flexible location model provides a considerably better fit (Fig A and Fig B in S3 Text). Thus, throughout this article, we choose to use the flexible location model and will vary the GBE associated parameter values. As illustrated in Fig 3, we consider a minimal chain length for the substrate (noted $L_{\text{min}}^{\text{GBE}}$) such that GBE is able to bind. We ensure that no daughter branch stands between the binding point and the non-reducing end of the branch. After binding, GBE cleaves at least $L_{\text{transferred}}^{\text{GBE}}$ glucose units. Finally, GBE must attach the cleaved chain on the initial substrate, following an intramolecular process, and creating a new A chain. To precisely describe this last step, we define two additional lengths. First, the new *α*-1,6 branching point must not be closer than $L_{\text{spacing}}^{\text{GBE}}$ from either the first glucose of the chain, or an *α*-1,6 branching point closer to the reducing end. Second, the new branching point must not be closer than $L_{\text{leftover}}^{\text{GBE}}$ from the non-reducing end of the substrate chain, which is the original position of cleavage. Thus, for a new branch to be created by GBE, the substrate branch must have a chain length greater than $L_{\text{min}}^{\text{GBE}}$, verifying:

$$\begin{array}{c} L_{\text{min}}^{\text{GBE}}>L_{\text{spacing}}^{\text{GBE}}+L_{\text{transferred}}^{\text{GBE}}+L_{\text{leftover}}^{\text{GBE}}. \end{array}$$ (1)

**Fig 4:**
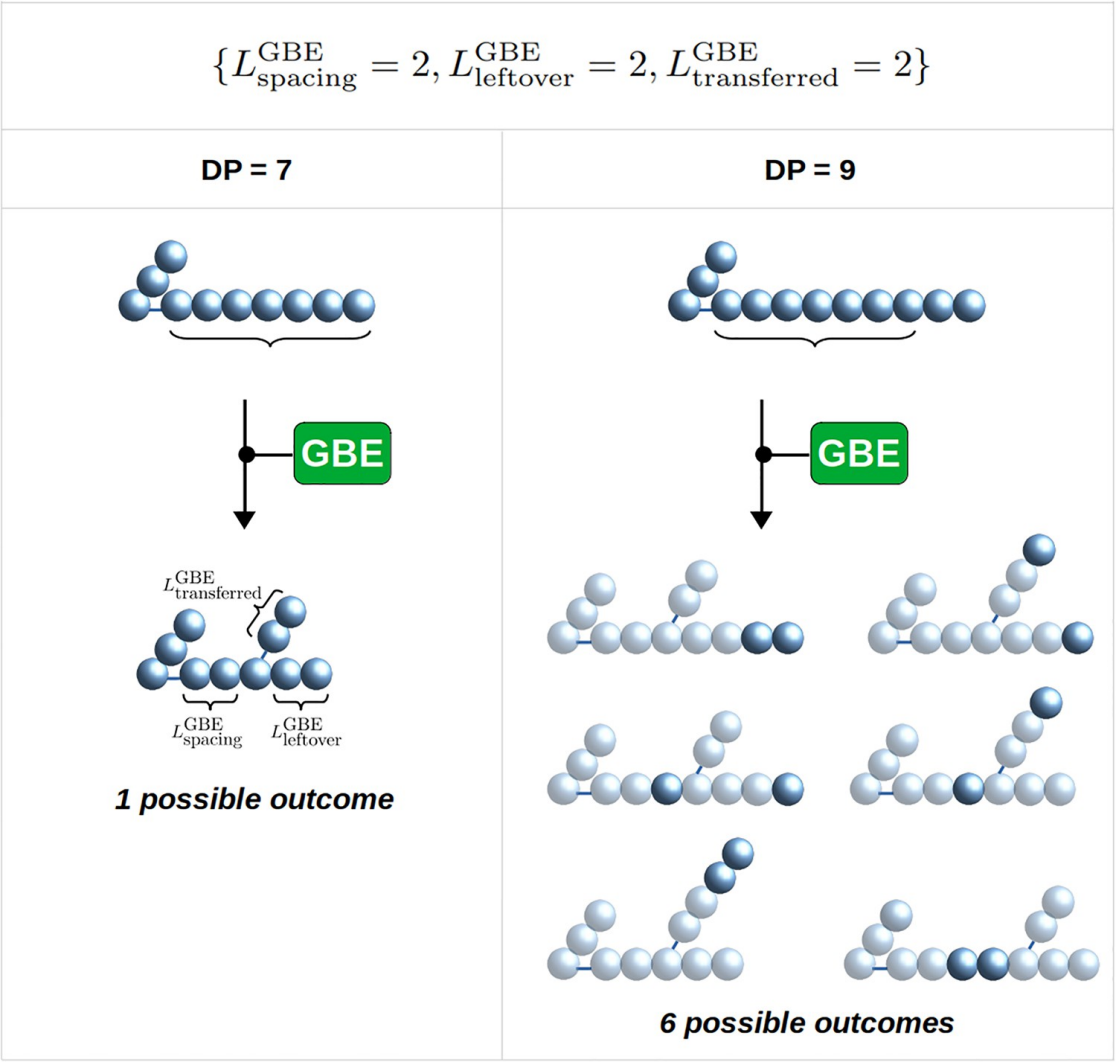
Illustration of the potential outcomes by GBE branching with $\left\{L_{\text{spacing}}^{\text{GBE}}=2,L_{\text{transferred}}^{\text{GBE}}=2,L_{\text{leftover}}^{\text{GBE}}=2\right\}$. With these minimal lengths, the minimal DP required for a branching to occur is DP = 7. If the chain length is longer, the number of possible outcomes increases. **Left:** With a substrate of DP = 7, only one outcome is possible. **Right:** With a substrate of DP = 9, up to 6 distinct outcomes are possible. Light grey represents the structure as it would be in case only one outcome would be possible, from a chain of DP equal 7.

To illustrate the impact of these minimal lengths on the reaction outcomes, in Fig 4 we detail the case of $\{L_{\text{spacing}}^{\text{GBE}}=2,L_{\text{transferred}}^{\text{GBE}}=2,L_{\text{leftover}}^{\text{GBE}}=2\}.$ If the substrate reaches a length of DP equal 7, the condition (Eq 1) is fulfilled and the reaction may take place. If this reaction occurs on a chain of minimal length, there is a single possible outcome (Fig 4, Left panel). If the branching occurs on a longer chain, several outcomes are possible, all fulfilling the set of rules specified by the triplet {2, 2, 2}. Fig 4 (Right panel) depicts the case of a substrate chain of DP equal 9, which results in 6 possible outcomes.

### 2.2 Analysis of the model’s properties with generic parameter values

#### 2.2.1 Elongation to branching ratio

**Fig 5:**
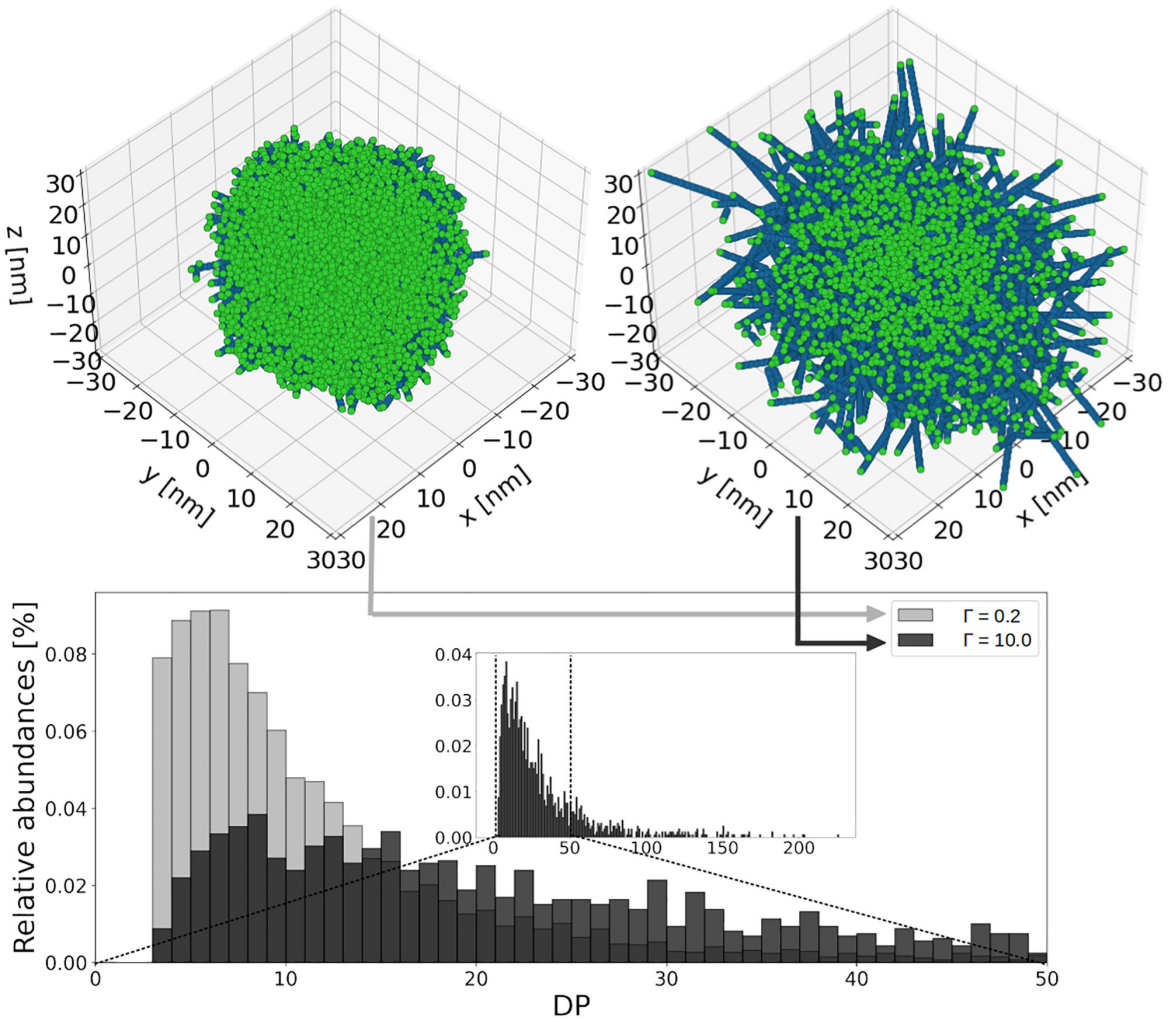
Simulated glycogen granule structures for different elongation to branching ratios (Γ). 50,000 glucose units are incorporated. **Top: 3D structures of glycogen granules.** Blue spheres represent the glucose units, green spheres the non-reducing ends. When Γ = 0.2, the structure of the granule is tightly packed. For Γ = 10.0, the structure of the granule is sparsely packed. **Bottom: Associated chain length distributions.** The light grey histogram shows the CLD for the tightly packed granule, while the black one shows that of the sparsely packed granule. The inset shows the full range of DP for Γ = 10.0. The longest chain is found to have a DP of 226.

When the simulation starts, the system is composed of a GN core with two primary chains standing in opposite directions in the center of the simulation space. Two enzymes (GS and GBE) modify the structure of the glycogen granule. To quantify their action, we define Γ as the ratio of the GS over GBE reaction rates. For Γ ≈ 0, branching dominates over elongation, and *vice versa* when Γ ≫ 1. Fig 5 (Top panel) shows two simulated glycogen structures obtained with Γ = 0.2 and Γ = 10, respectively. We observe that a low Γ corresponds to a tightly packed structure, while a high Γ leads to a sparsely packed structure, with further elongated chains. Both simulations have been performed with a high *N* value, such that the number of monomers is not a limiting factor. The simulations end when the total number of monomers incorporated into the growing granule is *N* = 50,000. As can be seen in Fig 5 (Top panel), when Γ increases, for the same number of glucose units incorporated into the granule, the length of the chains increases while their number decreases, and so does the number of non-reducing ends (represented by green spheres).

Fig 5 (Bottom panel) shows the chain length distribution (CLD) for the two simulated structures. It is computed as the abundance of each chain length, taking into consideration all the chains of the structure. With Γ = 0.2 (light grey histogram) the average DP is 12.2 with a peak in the range [4 − 8] DP. When elongation is stronger than branching (Γ = 10.0, black histogram) the distribution shifts to higher DPs, the mean DP is 38.8, and the intensity of the peak is much reduced. By analogy with the well-studied case of starch, a chain length distribution with abundant high DP, might be an indication of double helix formation [46]. We do not model double helices as such, but our results allow to determine the Γ range that might lead to double helix formation, and thus potential glycogen precipitation.

#### 2.2.2 Granule density

Our approach tracks the position (*x*, *y*, *z*) of each glucose unit in three-dimensions. This allows us to compute how densely the granules are packed. Granule packing is quantified by the occupancy Ω, which is defined by the volume occupied by glucoses *V*_glucose_ divided by the total volume *V*_total_,

$$\begin{array}{c} \Omega =\frac{V_{\text{glucose}}}{V_{\text{total}}}. \end{array}$$ (2)

**Fig 6:**
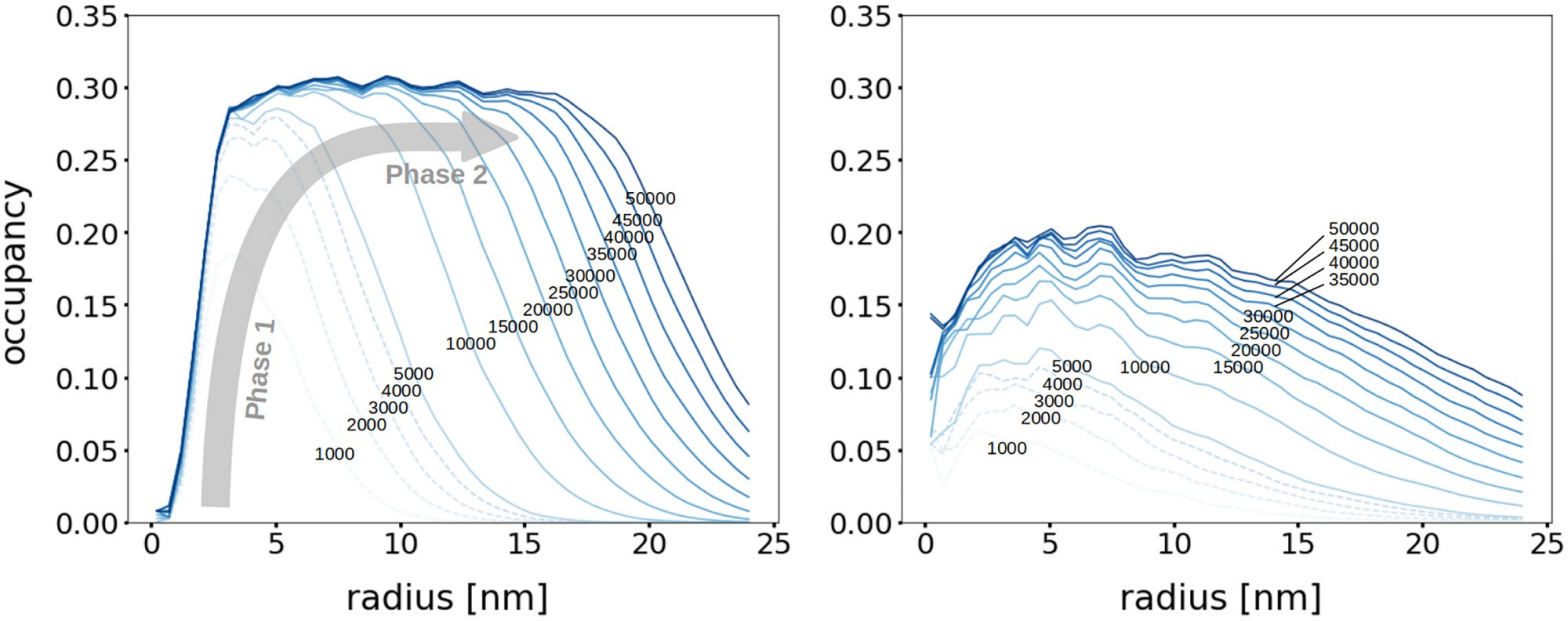
Dynamics of the occupancy profiles for a tightly (Γ = 0.2, Left) and sparsely (Γ = 10.0, Right) packed granule. Occupancy as a function of the radius at different simulation times. Each line corresponds to an incorporation of *N* = 5,000 glucose units. The simulation stops at *N* = 50,000. The grey arrow highlights the two phases of the granule synthesis dynamics. In phase 1, steric hindrance constrains are low, allowing occupancy to increase. In phase 2, i.e. after incorporation of ca. *N* = 10,000 glucose units, the occupancy reaches a plateau and the granule expands.

To characterise an entire granule, we consider the total volume *V*_total_ to be a sphere of gyration radius *R*_*g*_ (defined in the section Symbols definition). To determine the occupancy at a given radius *r* from the center of the granule, we estimate the local occupancy in a spherical shell between radii *r* and *r* + Δ*r*. Fig 6 displays how the occupancy Ω dynamically changes as a function of the radius *r* during granule synthesis, for two different values of the elongation to branching ratio Γ. The Left panel shows the formation of a tightly packed granule (Γ = 0.2), while the Right panel shows a sparsely packed structure (Γ = 10.0). Each line in the figure corresponds to the incorporation of 5,000 glucose units into the granule. It can be observed that granule synthesis proceeds in two phases. The first phase is characterised by an increase of the density close to the granule centre, while the radial extension increases only moderately. This can be explained by the fact that initially there is sufficient space to add new glucose units and there is hardly any steric hindrance among them. When steric hindrance starts to constrain the synthesis (after around 10,000 glucose units have been incorporated), the system transits to the second phase. The latter is characterised by a radial expansion of the overall structure, while the density remains approximately constant around 30%. The two phases can be observed for both Γ values considered, but they are more pronounced for the tightly packed granule (Γ = 0.2). Our simulated occupancy profile is in agreement with recent experimental measurements of the radial density profile of glycogen granules [37]. There, the authors also observed a rather constant density in the inner part of the granule, and a decrease at its periphery.

The relatively low occupancy close to the granule center is due to the presence of the GN core, which is not counted in the occupancy, but its corresponding volume cannot be filled with glucose units. We observe that at most 30% of the granule volume is occupied by glucose. This value is rather low, which is expected, since in the model, branches are straight helices, without any flexibility, while in reality, branches can bend and form locally higher densities. Specifically, this occupancy value is below Ω = 0.45, which we can estimate from previous studies [33]. There, the authors combine experimental and modelling approaches to conclude that granules of 55,000 glucoses have a radius of 21 nm [33]. Nonetheless, there are many uncertainties on the molecular masses experimentally measured. Thus, the occupancy value (i.e. Ω = 0.45) that we deduce from their work might hold large errors. For instance, it is unclear how the water and protein molecules embedded in the granules contribute to the molecular masses experimentally measured.

#### 2.2.3 Effect of the branching enzyme on the CLD

**Fig 7:**
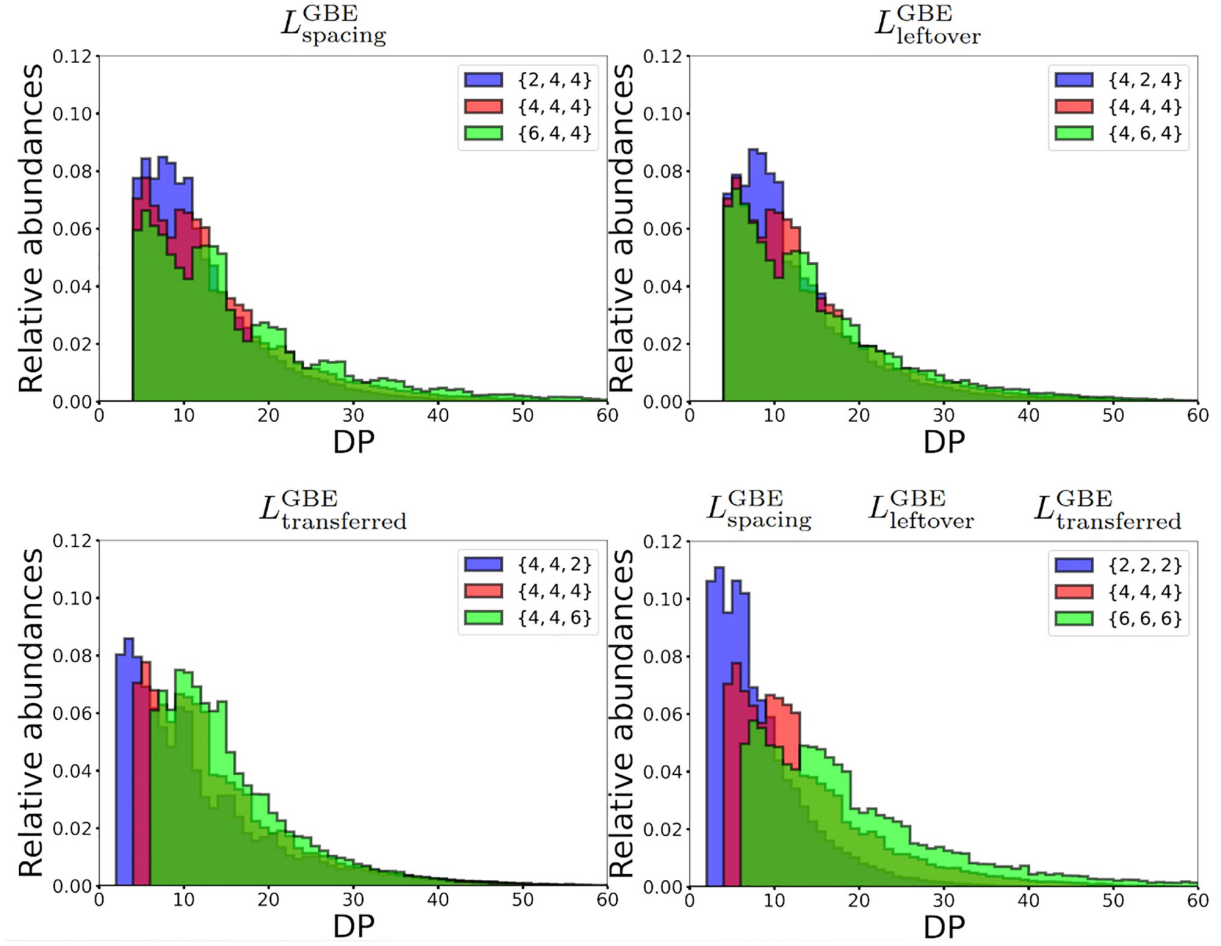
Effect of the minimal lengths of the branching mechanism on the CLD. **Top-left:** CLD for $L_{\text{spacing}}^{\text{GBE}}=2$, 4, and 6, respectively. When $L_{\text{spacing}}^{\text{GBE}}$ increases, a multi-modal distribution emerges. **Top-right:** CLD for $L_{\text{leftover}}^{\text{GBE}}=2$, 4, and 6, respectively. When $L_{\text{leftover}}^{\text{GBE}}$ increases, the peak is reduced and the overall distribution spreads towards higher DPs. **Bottom-left:** CLD for $L_{\text{transferred}}^{\text{GBE}}=2$, 4, and 6, respectively. When $L_{\text{transferred}}^{\text{GBE}}$ increases, the distribution shifts towards higher DPs. **Bottom-right:** CLD for $L_{\text{spacing}}^{\text{GBE}}=L_{\text{transferred}}^{\text{GBE}}=L_{\text{leftover}}^{\text{GBE}}=2$, 4, and 6, respectively. Varying these distinct minimal lengths concomitantly, combines the individual effects described above, when a single length is varied. Each CLD is the result of averaging 200 simulations of granules with 5,000 glucose units each.

The branching mechanism is characterised by a triplet of integer numbers, denoted $\left\{L_{\text{spacing}}^{\text{GBE}},L_{\text{transferred}}^{\text{GBE}},L_{\text{leftover}}^{\text{GBE}}\right\}$, which specifies a unique set of rules for the enzymatic reaction. These rules considerably impact the CLD. Fig 7 shows that when these minimal lengths increase, the peak of the CLD is less pronounced and the distribution spreads towards higher DPs. Also, a change in each minimal length has a specific effect on the CLD.

Increasing $L_{\text{spacing}}^{\text{GBE}}$ (Top-left panel) drastically reduces the peak and spreads the distribution, while making it bimodal. An increase in $L_{\text{spacing}}^{\text{GBE}}$ reduces the granule’s number of possible configurations. Fewer configurations are possible, and $L_{\text{spacing}}^{\text{GBE}}$ further shapes the chain length distribution. Chains of DPs that are combinations of $L_{\text{spacing}}^{\text{GBE}}$ and $L_{\text{leftover}}^{\text{GBE}}$ are favoured, resulting in local peaks. Increasing $L_{\text{leftover}}^{\text{GBE}}$ (Top-right panel) also decreases the peak and spreads the overall distribution towards higher DPs. $L_{\text{transferred}}^{\text{GBE}}$ (Bottom-left panel) has a different effect on the structure. Since $L_{\text{transferred}}^{\text{GBE}}$ is the smallest DP that can be formed, it is found on the leftmost part of the CLD, and variations in $L_{\text{transferred}}^{\text{GBE}}$ shift the overall distribution by the corresponding exact amount.

It is important to notice, that these results are obtained when branching dominates over elongation. Increasing the elongation to branching ratio Γ systematically smoothens any multi-modal CLD, because it introduces flexibility in the branching location. It also flattens the peak and spreads the distribution towards higher DPs. Consequently, bi-modal distributions are obtained for high $L_{\text{spacing}}^{\text{GBE}}$ and low Γ values.

As discussed in the Elongation to branching ratio section, we can compare the synthesis process of glycogen and starch. Specifically, in starch the CLD is bi-modal and shifted towards higher DPs as compared to glycogen [47]. Based on our preceding remarks, it could mean that starch branching enzymes are characterised by large substrate specificity lengths, corresponding to a more constrained mechanism than for glycogen. An alternative explanation for the arising of multi-modal CLDs, also based on highly constrained branching, is discussed in S2 Text.

### 2.3 Comparison to experimental data

#### 2.3.1 Parameter calibration

Based on our simulations, it is clear that the CLD is a signature of the branching enzyme mechanisms and substrate specificity. This signature results from the combination of many different parameters. Therefore, it is particularly difficult to analyse experimental data and infer parameter values without a complementary modelling approach. To extract useful information, and compare simulations to experimental data, we designed a fitting procedure, which is detailed in the Material and Methods section. With this strategy, we are able to determine parameter values that we compare to experimental results distinct from those used for the fitting.

GBE’s mechanism and substrate specificity are incompletely characterised, yet they drastically impact on the CLD, as shown in Fig 7. Therefore, we specifically focus on this enzyme. To this end, we use experimental data obtained by Sullivan et al. [25] for mice muscles, that we extracted from their publication using the software Engauge Digitizer [48]. In their article, after purification, the granule chains are debranched using isoamylase, and their degree of polymerisation is measured using size exclusion chromatography. Our fitting procedure can be applied to any glycogen data, obtained from any specie and tissue. The data by Sullivan et al. [25] present two major advantages for our study. First, they are quantitative measurements of good resolution. Second, the authors investigated the case of a defective GBE.

**Fig 8:**
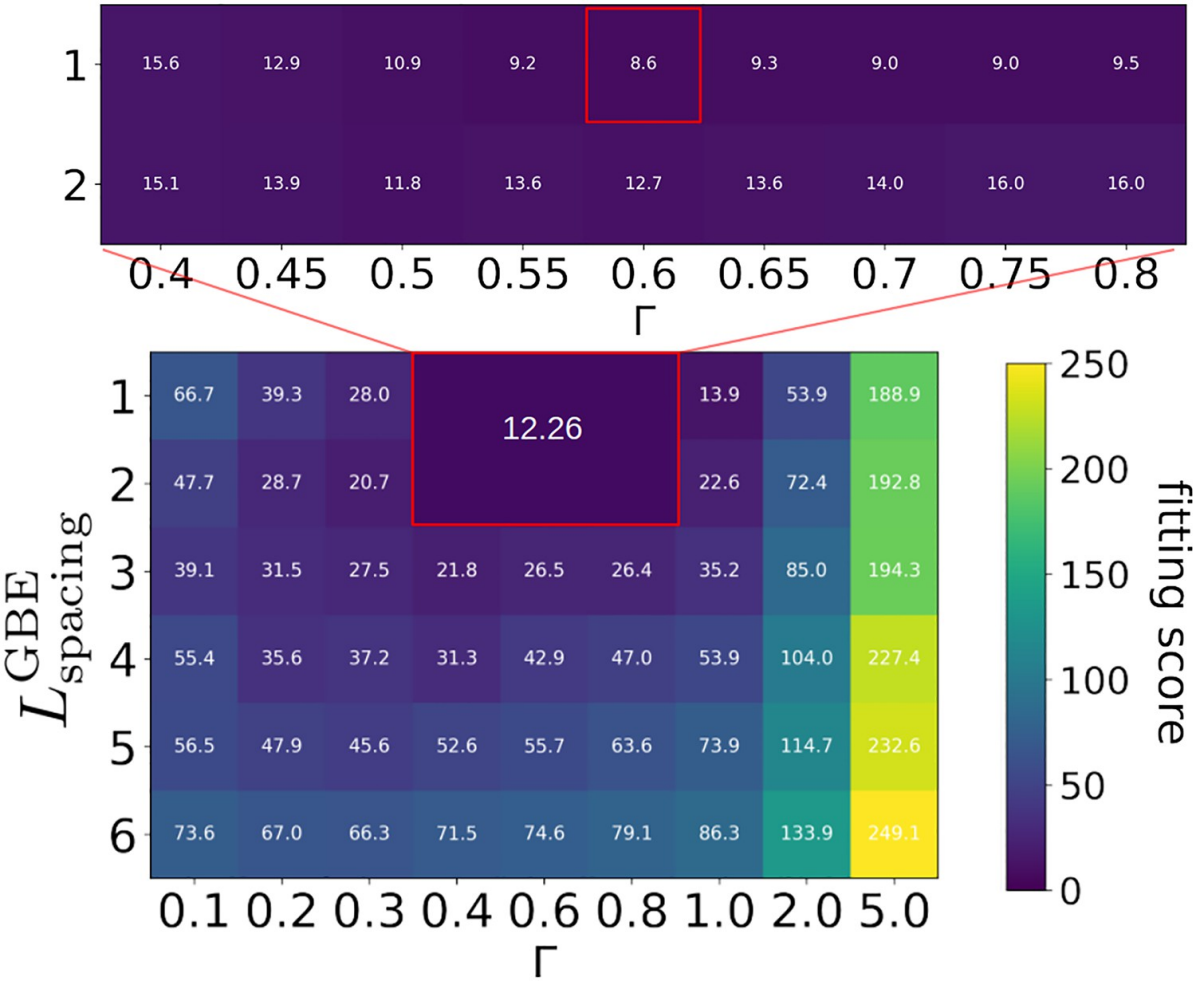
Heat-maps showing fitting scores for various sets of parameter values. The *Y*-axis shows $L_{\text{spacing}}^{\text{GBE}}$ ranging from 1 to 6. The *X*-axis shows the elongation to branching ratio Γ ranging from 0.1 to 5.0. A given cell corresponds to a set of parameter values {Γ, $L_{\text{spacing}}^{\text{GBE}}$, $L_{\text{leftover}}^{\text{GBE}}$, $L_{\text{transferred}}^{\text{GBE}}$, *ρ*}. Additional sets of parameter values are tested around good scores, i.e. the resolution on the elongation to branching ratio is increased, as well as the number of runs averaged. This area is surrounded by a red rectangle in which the average score is 12.26. Fitting scores are ranging from 8.6 to 249.1. The best score is 8.6 (red square in the inset heat-map) which corresponds to {Γ = 0.6, $L_{\text{spacing}}^{\text{GBE}}=1$, $L_{\text{transferred}}^{\text{GBE}}=L_{\text{leftover}}^{\text{GBE}}=3$, *ρ* = 0.65 nm}.

Fig 8 shows the heat-map containing the best fit we obtained with the model. Extensive scans of the parameter space (see Fig A and Fig B in S3 Text) have shown that best fits can be obtained for $L_{\text{transferred}}^{\text{GBE}}=L_{\text{leftover}}^{\text{GBE}}=3$. We therefore fix these two values in the following analysis. On Fig 8, the parameter $L_{\text{spacing}}^{\text{GBE}}$ is represented on the Y-axis, ranging from 1 to 6. The elongation to branching ratio Γ is varied on the X-axis. We notice that only low values for $L_{\text{spacing}}^{\text{GBE}},L_{\text{leftover}}^{\text{GBE}}\;\text{and}\;L_{\text{transferred}}^{\text{GBE}}$ allow to fit the experimental data. However, one should remember that these are minimal lengths and that the positions at which GBE is able to cleave and branch are flexible beyond these minimal lengths (see Fig 4). Overall, not only have we ran our fitting procedure using both the flexible and the strict location branching models, but also considering distinct values for *ρ* (Fig A and Fig B in S3 Text). Interestingly, the best fit is obtained for *ρ* = 0.65 nm, which reflects the realistic size of a glucose unit inside a helical chain. This highlights the role of the steric hindrance to mimic the granule’s structural properties. The best fit is obtained for the following set of parameters:

$$\{\Gamma =0.6,\;\;L_{\text{spacing}}^{\text{GBE}}=1,\;\;L_{\text{leftover}}^{\text{GBE}}=L_{\text{transferred}}^{\text{GBE}}=3\}.$$

Importantly, the parameter values for the branching enzyme inferred from the best fit are distinct from those reported in the field [33, 49, 50], especially for the typical spacing observed between two branches [33]. Also, based on our results, GBE is able to transfer less than 4 glucose units. Knowing that GS’s chain length specificity has to follow the same rule, this questions the commonly assumed value of DP4 as the minimal length that can be elongated by GS.

**Fig 9:**
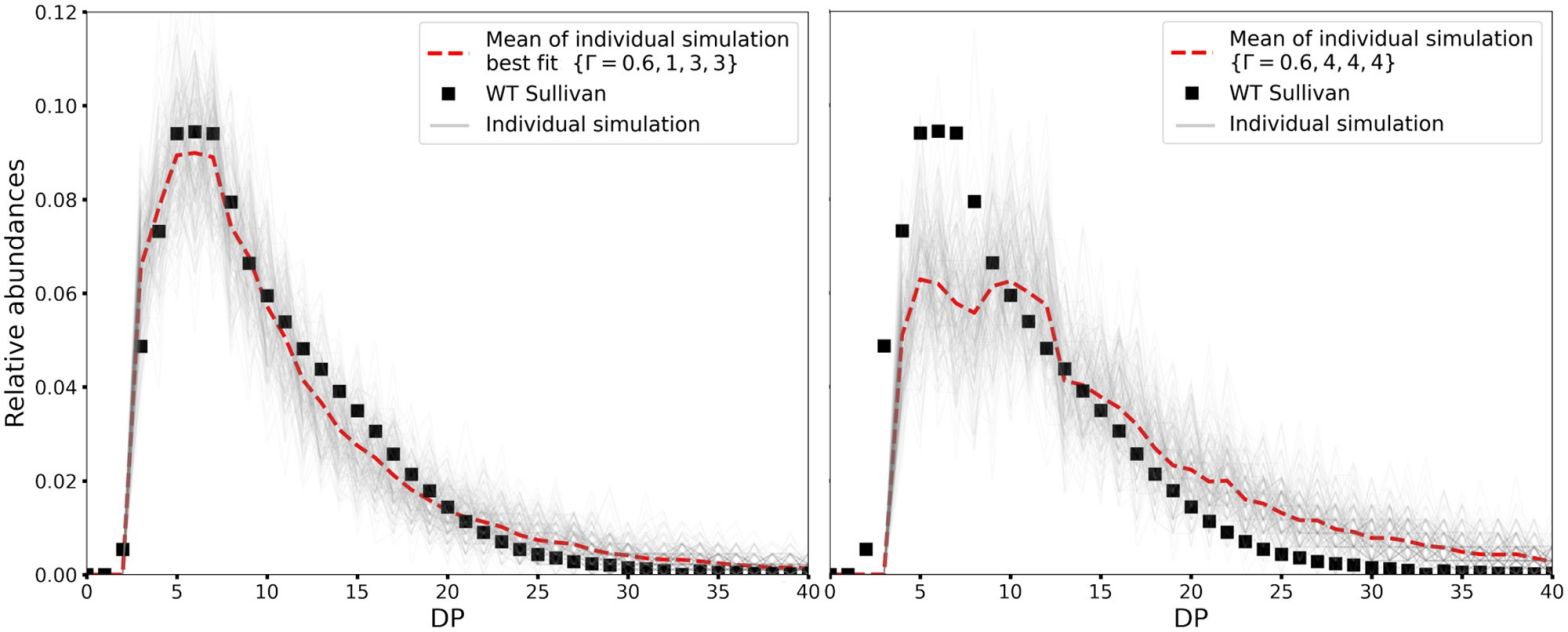
Comparison of simulated *versus* experimental CLDs. Experimental data are from Sullivan and coworkers [25] (black squares). In each simulation run 50,000 glucose units are incorporated in the growing granule (grey line). The average over 200 runs is represented as a red dotted line. **Left:** The CLD for the best fitting score (S=8.6) is obtained with {Γ = 0.6, $L_{\text{spacing}}^{\text{GBE}}=1$, $L_{\text{leftover}}^{\text{GBE}}=3$, $L_{\text{transferred}}^{\text{GBE}}=3$, *ρ* = 0.65 nm}. Our best fit almost perfectly captures the experimental CLD. **Right:** CLD using parameter values typically assumed in the literature {Γ = 0.6, $L_{\text{spacing}}^{\text{GBE}}=4$, $L_{\text{leftover}}^{\text{GBE}}=4$, $L_{\text{transferred}}^{\text{GBE}}=4$, *ρ* = 0.65 nm} [49, 50]. The simulated CLD differs a lot from the experimental one, with under-representation of small DPs, and over-representation of high ones.

In Fig 9, the CLD for the best fit is shown, together with simulation results for parameter values typically reported in the experimental literature, i.e. $\left\{L_{\text{spacing}}^{\text{GBE}}=4,\;L_{\text{leftover}}^{\text{GBE}}=4,L_{\text{transferred}}^{\text{GBE}}=4\right\}$. For the latter, we observe a plateau from DP4 to DP10, while experimental data show a peak between DP6 and DP8. Additionally, longer chains (DP ≥15) are over-represented. Noticeably, for this set of GBE minimal lengths, our model is not able to reproduce the experimental data by Sullivan and coworkers [25], even when varying the elongation to branching ratio Γ (see Fig A in S3 Text).

#### 2.3.2 Glycogen structure using the fitted parameters

In this section, unless otherwise specified, we assume that GBE’s parameter values are set to those of the best fit $\left\{\Gamma =0.6,\;\;L_{\text{spacing}}^{\text{GBE}}=1,\;\;L_{\text{leftover}}^{\text{GBE}}=L_{\text{transferred}}^{\text{GBE}}=3\right\}$. With those, we simulate the synthesis of glycogen granules, and compute their structural features and macroscopic characteristics (see Table 1). For each of those, the average values and standard errors are calculated over 30 granules with *N* = 50,000 glucose units. The number of non-reducing ends (noted *N*_NREs_) is equal to the total number of chains, since there is one non-reducing end per chain.

**Table 1:** Summary of the granule structural features and macroscopic characteristics.

	Structural features					Macroscopic characteristics	
	*N*_NREs_	A:B	CL	Branching degree	Generation	Ω	*R*_*g*_
Γ = 0.6	4,135.1 ± 57.6	0.98 ± 0.02	12.09 ± 0.17	0.0901 ±0.0014	21.8 ± 1.4	0.521 ± 0.012	19.42 nm ± 0.15
Γ = 50.0	430.9 ± 21.5	1.03 ± 0.06	116.32 ± 5.95	0.0087 ± 0.0004	11.3 ± 1.3	0.025 ± 0.007	54.83 nm ± 7.69

In the case of the best fit, for Γ = 0.6, we find that on average, granules are made of ca. 4,135 chains, with a chain length of 12.09 glucoses. These two quantities are intrinsically linked since the total number of glucoses is fixed to *N* = 50,000. The higher the average chain length (noted *CL*), the lower the total number of non-reducing ends, since *CL* ⋅ *N*_NREs_ = *N*. The A:B ratio is found to be 0.98. With *N*_NREs_ = 4, 135, it means that ca. 2,047 chains are of type A, while 2, 088 are of type B. The branching degree is found to be 9.01 %. This is in good agreement with the range typically reported in glycogen (i.e. [5–10 %] [9, 29]), while starch exhibits much lower values (typically between 1 and 5 % [26–28]). The average occupancy is Ω = 0.521, which means that half of the total granule volume is filled with glucoses. The gyration radius is *R*_*g*_ = 19.42 nm which is consistent with radii reported for big *β* granules (molecular weight ca. 10^7^g.mol^−1^), like the ones considered here (*N* = 50,000) [13, 51].

It is interesting to notice that ca. 21 nm is the upper limit for the radius of a glycogen granule of ca. 50,000 glucoses, when considering the fractal structure depicted by Meléndez et al. [6]. Thus, with our approach, that is not based on a fractal structure, we determine a gyration radius close to their results. However, the glycogen structures we simulate are deeply distinct from theirs. In our simulations, we observe that the density is approximately constant with the radius, while a fractal glycogen granule has a density that increases exponentially with the radius, so resembling a rather empty shell. Also, we show that our simulations can reproduce characteristic quantities (summarised in Table 1 for Γ = 0.6), which are in good agreement with experimental results. Thus, our model appears to provide a more realistic depiction of glycogen granules.

For comparison, in Table 1, we also report the case of Γ = 50.0, while keeping all other parameter values unchanged. By increasing Γ, we force elongation over branching. As expected, less chains are created but they are longer. Consistently, the branching degree is also lower. In our model, chains are rigid rods that cannot bend, which leads to a bigger radius of gyration. Since the total content of glucose is the same like for Γ = 0.6, the occupancy Ω decreases almost proportionally. *In vivo*, glycogen granules might follow a distinct behaviour, where the presence of long chains could trigger precipitation, like for instance reported in the Lafora and GBE diseases [25, 52].

The formation of a new A chain results from branching either an A or a B chain. These different events can be represented as reactions and described as follows:

$$\begin{array}{c} R_{A\to AB}:\;\;\text{A}\overset{\text{GBE}}{\to }\text{B}+{\text{A}}_{\text{new}},\\ R_{B\to AB}:\;\;\text{B}\overset{\text{GBE}}{\to }\text{B}+{\text{A}}_{\text{new}}. \end{array}$$

**Fig 10:**
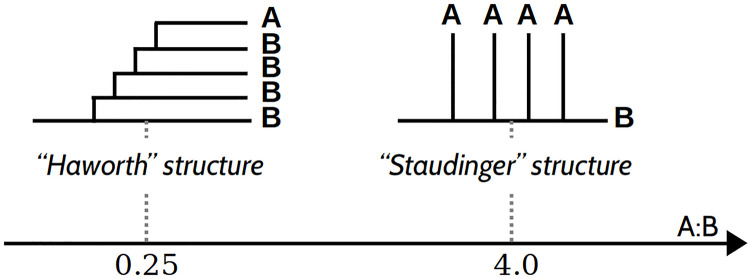
Two schematic branched structures with different A:B ratios. For a “Haworth”-like structure, branching reactions tend to occur on A chains, leading to a low A:B ratio, while for a “Staudinger”-like structure, branching on B chains is favoured, leading to a high A:B ratio.

To illustrate how different branch structures correspond to specific A:B ratios, we sketch extreme cases in Fig 10. If the *R*_*B*→*AB*_ and *R*_*A*→*AB*_ reactions are equally likely, a purely probabilistic approach predicts that, on average, the A:B ratio is equal to 1, independently of the relative activities of the enzymes (see Fig A in S4 Text). Such a ratio is observed in Table 1, with A:B = 0.98 ± 0.02 and A:B = 1.03 ± 0.06, which is in good agreement with reported values for glycogen (A:B in the range 0.6 − 1.2 [31]). Other closely related branched polysaccharides can exhibit different A:B ratios. For example, amylopectin has a typical A:B ratio in the range from 1.5 to 2.6 [31, 32], depending on the organism. It is therefore interesting to investigate which factors influence this ratio.

Here, we identify two effects of the interplay between the branching mechanism and the dynamics of the granule structure, highlighting once again the importance of the branching enzyme on the emerging structural patterns. First, considering a small Γ, branching dominates over elongation, and the enzyme branches as soon as it can. Thus, if in addition $L_{\text{spacing}}^{\text{GBE}}$ is small, the A chains are closely stacked together and the granule is tightly packed. In this regime, overall, A chains are closer to other A chains than to B chains. Due to the increased steric hindrance for A chains, B chains react more easily, which favours the *R*_*B*→*AB*_ reaction, and leaves A chains unchanged. Therefore, the A:B ratio increases, meaning that it shifts to the right along the axis in Fig 10. Second, if $L_{\text{transferred}}^{\text{GBE}}>L_{\text{leftover}}^{\text{GBE}}$, when a branching occurs, on either an A or a B chain, the newly formed A chain will on average be longer than the B (leftover) one. Therefore, the new A chain will react faster, favouring *R*_*A*→*AB*_, and lead to a reduction of the A:B ratio, meaning that it shifts to the left along the axis in Fig 10. We confirmed this effect for the specific case of $L_{\text{transferred}}^{\text{GBE}}=7$ and $L_{\text{leftover}}^{\text{GBE}}=3$. The resulting structures have an A:B ratio on average equal to 0.80 ± 0.02. If the values of the minimal lengths are exchanged, such that $L_{\text{transferred}}^{\text{GBE}}=3$ and $L_{\text{leftover}}^{\text{GBE}}=7$, the A:B ratio is instead equal to 2.01 ± 0.04. It appears that the $L_{\text{transferred}}^{\text{GBE}}$ and $L_{\text{leftover}}^{\text{GBE}}$ are specific mechanistic features that uniquely determine the A:B ratio. Consequently, they could cause the main structural differences observed between amylopectin and glycogen.

## 3 Material and methods

### 3.1 Simulation procedure

We present a model that simulates the dynamics of glycogen synthesis. We record all enzymatic reaction steps, the time at which they occur, and the full three-dimensional details of the glycogen structure at any time point. The model specifically keeps track of each glucose position, the complete connectivity of the chains, and the position of each branching point. To account for this complexity, we implement the model using a stochastic algorithm. This approach also allows to specifically consider how changes in the glycogen structure enable or disable enzymatic reactions. For comparison, employing a deterministic approach, for instance based on systems of ordinary differential equations, would lead to unnecessarily complex simulation rules, that would also include additional ad-hoc assumptions. Besides, randomness has a stronger impact on a system as it involves small numbers, like the one we consider here. Indeed, the synthesis and breakdown of a single glycogen granule of ca. 50,000 glucose units involves only a small number of enzymes. For example, experiments indicate that on average a single glycogen synthase enzyme is active per granule [53]. As a result, a stochastic approach appears very natural to deal with this complex and spatially structured substrate.

We base our simulations on the Gillespie algorithm [54, 55]. At each time step, it consists in randomly selecting both an enzymatic reaction and its duration, while systematically updating the structure of the glycogen molecule accordingly. Although the enzymatic reaction is selected from a uniform distribution, the time is chosen from an exponential distribution, such that the Gillespie algorithm allows to simulate the real time of the dynamics of the system, as far as the underlying enzymatic mechanisms and their kinetic parameter values are known. Each reaction is associated to a specific propensity, that we choose as proportional to the concentration of enzymes and substrate, thereby following a typical mass-action kinetics.

The Gillespie algorithm accounts for all possible reactions, and keeps track of any modification of the granule structure, such that only possible reactions can be selected. Nonetheless, because of the complexity of the structure we simulate, to account for steric hindrance among different branches, we instead allow for certain reactions to be rejected. For these rejected reactions, the time elapsed is not accounted.

We provide details about the Gillespie algorithm (Fig A in S5 Text), and how it was employed to simulate the dynamic changes of the three-dimensional structure of the granule (Fig B in S5 Text). Moreover, the source code of our model, together with Jupyter Notebooks that recreate the main figures of this manuscript, are available on our gitlab repository (link provided below under Data availability).

### 3.2 Best fit algorithm

The model contains various kinds of parameters. Some describe the physical properties of the glycogen structure, others relate to the enzymatic activity, including the enzyme substrate specificities. On the one hand, certain parameter values are inferred from literature data, for instance the minimum DP for GS to act ($L_{\text{min}}^{\text{GS}}$). On the other hand, other parameter values are free to be fitted by our custom designed best fit algorithm. Here we choose to fit the minimum DP between two branches after a branching reaction ($L_{\text{spacing}}^{\text{GBE}}$), the minimum DP between the non-reducing end of the mother branch and the new branching point ($L_{\text{leftover}}^{\text{GBE}}$), and the ratio between the elongation and branching reaction rates (Γ).

Our best fit algorithm consists in setting bounding ranges for the values of the parameters to be fitted, and systematically scanning the parameter space thereby defined. For each set of parameter values tested, we run 50 simulations, take the average of the resulting CLDs, and compare it to our targeted experimental data. For each set of parameter values, the comparison to the experimental data is measured by the score S, defined as follows:

$$\begin{array}{c} S=\sum_{\text{DP}=1}^{n}{\left(A_{\text{exp}}\left(\text{DP}\right)-{\bar{A}}_{\text{sim}}\left(\text{DP}\right)\right)}^{2}, \end{array}$$

where $A_{\text{exp}}\left(\text{DP}\right)$ is the experimental CLD abundance for a given DP and ${\bar{A}}_{\text{sim}}\left(\text{DP}\right)$ the average simulated CLD abundance for a given DP. Therefore, S measures the squared difference between the average simulated and the experimental CLDs. The best fit is found for the set of parameter values that has the lowest score S.

## 4 Conclusion

### 4.1 Discussion of the results

According to the World Health Organization, metabolic diseases are a burden in western countries. Among them, Glycogen Storage Diseases (GSDs), Lafora disease, Adult Polyglucosan Body Disease (APBD), and even diabetes directly or indirectly involve glycogen. Investigating the regulation of glycogen’s structural properties could therefore strongly contribute to further understand such diseases. Computational models that encompass the complex interplay of both glycogen’s structure and its metabolism, allow to tackle this challenge but remain poorly exploited.

In this article, we present a stochastic structural model for glycogen synthesis. The model provides a precise description of both the structure of glycogen in three dimensions, that we can visualise, and the detailed dynamics and mechanistics of the underlying enzymatic reactions. For instance, both elongation and branching have precise rules regarding their substrate specificities. Modelling glycogen granules in three dimensions is made possible by a coarse-grained geometrical description at the glucose level, allowing us to track all the glucose units in space. This description also accounts for the steric hindrance effects resulting from the impossibility for the chains to overlap. Simulating reaction dynamics relies on a Gillespie algorithm, which determines when and where a reaction of branching or elongation takes place, thereby dictating the corresponding change in the three-dimensional structure.

We show qualitatively how enzyme activity affects glycogen structure for generic sets of parameter values. We highlight two different synthesis regimes, depending on Γ, the ratio between the elongation and branching reaction rates. By varying this ratio, either small and dense, or big and sparse granules can be simulated. *In vivo*, it can be expected that Γ depends on numerous factors, such as the organism under investigation, the cell type, and possibly the external conditions. In addition, a model that would consider chains bending and intermolecular interactions might lead to more complex results, in particular during the synthesis of sparse granules. In our results, the phenomenology of the two synthesis regimes is also confirmed by the occupancy profiles along the radius of the corresponding granules. In both synthesis regimes, first the center of the granule is filled around GN, before reaching a critical density, preventing further internal reactions to occur due to steric hindrance. Then, the granule expands such that the density remains approximately constant within the sphere defined by the gyration radius. While this result is a consequence of the geometrical assumptions of our model, it is interesting to note that a glycogen fractal description would instead give a density exponentially increasing with the granule radius. Besides our own results and the various arguments exhibited against a fractal representation of glycogen, we would like to highlight here that, as soon as one considers even a single degree of freedom in the glycogen branching reaction, it would rapidly lead to the loss of any “fractal-like” structure. Such degrees of freedom are necessarily present in natural conditions. For instance, the dihedral angles defining the *α* − 1, 6 bonds may take various values, making a fractal pattern very unlikely *in vivo*. Beyond these spatial considerations, the model establishes a natural and clear connection between enzymes’ reaction rates, and both the degree of polymerisation of the chains, and the number of non-reducing ends.

Our results show that the chain length distribution of glycogen is highly sensitive to the branching reaction, predominantly its mechanism. Each of the three characteristic minimal lengths of the reaction has specific effects on the CLD. Additionally, if any of them increases, less branching outcomes are possible, eventually leading to a bi-modal or even multi-modal distribution. This effect is enhanced by high branching activity. When varied together, these minimal lengths show even more complex imprints on the CLD. In contrast, multi-modal distributions become less pronounced by increasing the elongation reaction rate, because rapid elongation increases the number of possible configuration outcomes. Additionally, increasing elongation leads to longer chains and results in a CLD spreading towards higher DPs. Altogether, we show that the CLD, and in particular peaks’ location and intensity, are subtly affected by several complex effects.

Guided by these findings, we propose to consider the CLD not only as an important structural feature of glycogen, but also as a signature of GBE. Thus, fitting experimental data with our model arises as a natural strategy to infer knowledge on the GBE mechanism. Not only did we illustrate the strength of our fitting procedure on an experimental data set from Sullivan et al. [25], but we also extracted several macroscopic characteristic features from the resulting fit, which we compared to various literature sources, thereby confirming the validity of our results. Using this fit, at the microscopic level, we were able to discriminate between the two branching models we hypothesised, and selected the flexible location branching over the strict one. Besides, we could critically evaluate parameter values typically reported in the literature. For instance, it is often assumed that GBE transfers branches of DP 4 or 6, or even longer [33, 49, 50]. Similarly, it is typically reported that around 6 glucose units space two branches. Within the framework of our model and its underlying assumptions, it is impossible to reproduce CLDs of *in vivo* glycogen using the above mentioned values. Instead, our fitting procedure suggests that a high flexibility is necessary for both the branching mechanism and the minimal lengths involved. This finding fully confirms the importance of modelling glycogen synthesis using a stochastic approach.

Moreover, our customisable branching model shows that the A:B ratio is independent of the kinetic parameter Γ, but specifically determined by the difference between $L_{\text{transferred}}^{\text{GBE}}$ and $L_{\text{leftover}}^{\text{GBE}}$. Based on these observations, we hypothesise that the branching mechanism is chiefly responsible for the structural differences observed between starch and glycogen.

Throughout this study, our coarse-grained approach accounts for the contribution of individual glucoses to the overall granule structure, by considering them as spheres of radius 0.65 nm. It is interesting to notice that, if we set the glucose volume to 0 nm^3^, the CLD remains almost unchanged while other macroscopic properties of the granule, such as its overall volume, are dramatically impacted. This observation confirms once more that the glycogen CLD is primarily shaped by the enzyme mechanisms.

### 4.2 Outlook

It is important to keep in mind that our model contains limitations that may be circumvented by further refining the model assumptions. In our model, the simplified coarse-grained representation of glucoses assumes that all of them are arranged in single helices. This hypothesis implies that all *α* − 1, 4 glycosidic bonds have the same angle values. Yet, *in vivo*, this is highly unlikely, and instead, the dihedral angles of the *α* − 1, 4 bonds should be able to take various values. To take this into account, we could randomly pick the dihedral angles of the *α* − 1, 4 bonds using the Ramachandran plots of their energetically favourable regions. As a first trial, we could use that of maltose, that is well characterised [56]. We expect that, by introducing such disorder in the angles, chains will appear longer. Besides, in the model, we consider that all chains are stiff. Thus, to improve the macroscopic representation of the chains, we could introduce the possibility for them to bend when encountering steric hindrance. To do so, we would minimise their torsion energy, like it is done in polymer physics models [57]. In contrast to the suggestion for introducing variability in the *α* − 1, 4 bond, accounting for the flexibility of the chains might lead to a higher granule density, and thus, a lower radius. The fact that these two effects might cancel each other, possibly explains why our simplified model is nonetheless able to capture realistic *in vivo* granule radii. Besides, in abnormal conditions, the potential formation of double helices may not only lead to glycogen precipitation, but also prevent enzymatic reactions. Thus, a later improvement of the model could include to tune the enzymes’ reaction rate depending on the substrate chain configuration and length.

Throughout this article, we focus on glycogen synthesis, yet, simulating the degradation dynamics with the algorithms we developed would be straightforward. We expect residual degradation activity to only lightly modify the effective elongation to branching ratio Γ, and slow down the synthesis. In such a case, the CLD would be slightly shifted to the left, and the first chain length detected would be the smallest value between $L_{\text{transferred}}^{\text{GBE}}$ and its GP counterpart. It could be particularly interesting to account for the degrading enzymes, beyond a residual activity, in case the synthesis is defective. Indeed, abnormal structures produced by defective synthesis enzymes could be degraded, and thereby corrected, by degrading enzymes. For instance, if $L_{\text{transferred}}^{\text{GBE}}$ becomes too short for GS to elongate the newly formed chain, GDE could unbranch the latter and thereby preserve the macroscopic properties of the granule. Last, following an approach analogous to the one taken in this article, one could choose to investigate the glycogen granules’ breakdown in full depth, by first synthesising granules and then proceeding to their degradation. Although, for the sake of simplicity, we would then uncouple in time the synthesis and degradation processes, it would still be very interesting to study the mutual impact of distinct modes of synthesis and degradation on the overall glucose release and fixation.

In this article, we show that the enzyme substrate specificity strongly influences the enzyme activity, leading to distinct chain length distributions and number of non-reducing ends. Noticeably, other modelling approaches, such as kinetic models using systems of ordinary differential equations (ODEs), instead consider glycogen as a single metabolite, approximated by the sum of all glucose units that compose it. A direct consequence is that any structural aspects are neglected and these models cannot differentiate between a single chain of 50,000 glucoses, and an actual granule of the same weight. Still, for instance, the number of non-reducing ends available for elongation are drastically different in these two cases. Thus, coupling our model to glycogen metabolic ODE models, would constitute a hybrid approach that would include key structural details, while enlarging its biochemical scope. It would thereby open up a whole new range of modelling possibilities. For instance, it would allow to investigate the interplay between glycogen structure and the evolution in time of important metabolites (e.g. glucose-1-phosphate, glucose-6-phosphate, and UDP-glucose), under various physiological conditions, including diseases scenarios. Using this approach, we shall be able to characterise the phenomenology of each glycogen storage disease, with a focus on the role of glycogen structure, and address questions such as glycogen accumulation, glucose cycling, glucose homeostasis, and even glycogen precipitation.

## Symbols definition

**Table:** 

*α* and *β*	Different forms of glycogen granules as described by [13]. The first one being aggregates of the second.
*ρ*	Radius of a monomer unit described as a sphere in the model.
*l*	Distance between two overlapping spheres.
*N*	Number of glucose units incorporated in a granule.
$L_{\text{spacing}}^{\text{GBE}}$	Minimum distance (in glucose units) between the newly formed chain and its closest sister chain after branching.
$L_{\text{transferred}}^{\text{GBE}}$	Minimum chain length (in glucose units) cleaved and transferred.
$L_{\text{leftover}}^{\text{GBE}}$	Minimum distance (in glucose units) between the non-reducing end of the mother chain and the newly created branching point.
Γ	Ratio of the elongation and branching reaction rates.
*R*_*g*_	Radius of gyration (i.e. radius of a glycogen granule in nm) $R_{g}=\sqrt{\frac{1}{N}\sum_{i=1}^{N}{\left(\vec{r_{i}}-\vec{r_{\text{mean}}}\right)}^{2}},$ where *N* is the total number of glucose units in the granule, *r*_*i*_ the spatial coordinate of the *i*^*th*^ glucose unit, and *r*_mean_ that of the center of mass of the granule.
*A* : *B*	Ratio of the number of A chains (that do not carry any daughter chain) over B chains (that carry at least one).
*V*_glucose_	Volume effectively occupied by all the glucose units incorporated in the granule.
*V*_tot_	Volume of a glycogen granule (i.e. $4/3\cdot \pi \cdot {R_{g}}^{3}$).
Ω	Occupancy, defined as the ratio of *V*_glucose_ over *V*_tot_.
S	Score to measure the squared distance between simulated and experimental CLDs, defined as the mean square difference between the two curves.

## Supporting information

S1 Text Effect of self-exclusion. Chain length distributions obtained for different monomer sizes, showing the effect of steric hindrance on the structure of glycogen. 3 scenarios are discussed.
(PDF)

S2 Text Flexible location *versus* strict location branching models. Two models for the branching mechanism of GBE are proposed and discussed.
(PDF)

S3 Text Scope of the parameter space. The scope of the parameter space searched to find the best set of parameter values is presented. The latter minimises the mean square error between the experimental and the simulated CLDs. Two branching models are compared, resulting in heatmaps with corresponding fitting scores.
(PDF)

S4 Text Probabilistic approach to the A:B ratio. Discussion about the expected value for the A:B ratio in a purely probabilistic approach.
(PDF)

S5 Text Numerical procedures. Explanation of the Gillespie direct method used to simulate the glycogen synthesis reactions. Flow diagram showing the main steps of the simulations.
(PDF)
