# Supplementary material for: Stochastic modelling of a three-dimensional glycogen granule synthesis and impact of the branching enzyme
Source: PLoS Comput Biol. 2023 May 19;19(5):e1010694. doi: 10.1371/journal.pcbi.1010694 (PMC10198547; doi:10.1371/journal.pcbi.1010694)
Supplement: S1 Text — Chain length distributions obtained for different monomer sizes, showing the effect of steric hindrance on the structure of glycogen. 3 scenarios are discussed. (PDF) [file pcbi.1010694.s001.pdf]

S1: Effect of self-exclusion

Modelling the 3D structure of glycogen, while considering self-exclusion among chains, allows investigating glycogen chain length distributions, molecular density, size of the granules, and crowding at the surface. But is this detailed approach strictly required for studying glycogen's macroscopic properties? In the following, we test various radius values for the spheric description of the glucose units and look whether they affect the chain length distribution, a key macroscopic property of glycogen.

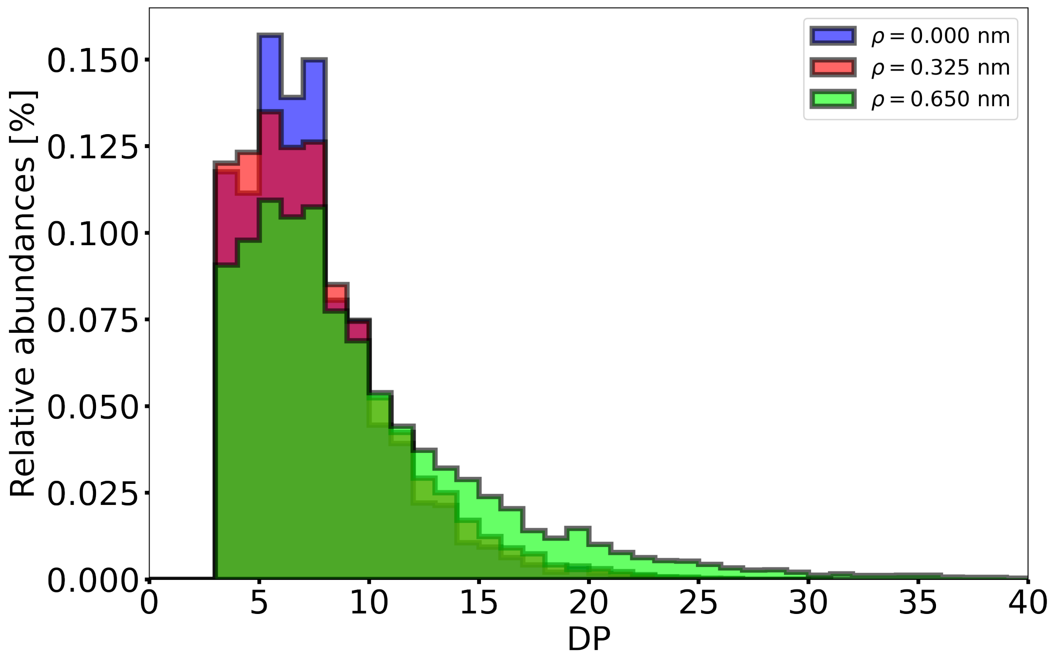

Fig A. Effect of the steric hindrance on the CLD. Chain length distributions for $\rho = 0$ nm (blue), $\rho = 0.325$ nm (red), and $\rho = 0.650$ nm (green). For $\rho = 0$ nm, the distribution exhibits a higher peak at DP [6-8] than for $\rho = 0.325$ nm and $\rho = 0.650$ nm. Opposite, for $\rho = 0.325$ nm and $\rho = 0.650$ nm, the higher DPs (from DP 12) are over-represented as compared to the case of $\rho = 0$ nm.

Three scenarios are investigated: $\rho = 0$ nm, $\rho = 0.325$ nm, and $\rho = 0.650$ nm. For $\rho = 0$ nm, the glucose units have no volume and thus no steric hindrance arises, which allows chains to overlap. $\rho = 0.325$ nm is equal to half of the helix's van der Waals radius, while $\rho = 0.650$ nm is its total radius. Fig A shows that short chains are more abundant for $\rho = 0$ nm than for the other two values of ρ. Opposite, long chains are more abundant for non-zero ρ. As soon as $\rho > 0$ nm, since elongation involves adding a single glucose unit, while branching means transferring an entire piece of a branch, it is easier to find the necessary space around the substrate for allowing elongation to take place as

compared to branching. This is reinforced by the fact that when adding a new glucose unit at the non-reducing end of a branch, we do not elongate the substrate by the total length of a glucose unit, but only its radial contribution, which is $l = 0.24$ nm (see Fig 2 of the article). As a consequence, steric hindrance stronger impacts branching than elongation. In other words, the number of branching attempts rejected due to steric hindrance is higher than that of elongation. If we would define effective branching and elongation rates that respectively account for these rejections, the branching effective rate would reduce much more than that of elongation. This would lead to an effective elongation to branching ratio Γ_{eff} which would increase with the effect of steric hindrance. In the section Elongation to branching ratio, we concluded that as Γ increases, the overall CLD shifts towards higher DPs and the distribution peak decreases. $\Gamma_{\text{eff}}^{\rho=0} < \Gamma_{\text{eff}}^{\rho=0.325} < \Gamma_{\text{eff}}^{\rho=0.650}$ therefore explains both the over-representation of high DPs and the peak reduction as ρ increases in Fig A. Despite these changes in the CLD, as the steric hindrance increases, we can remark that not only is the number of peak conserved (here unimodality) but their location too.
