# Supplementary material for: Stochastic modelling of a three-dimensional glycogen granule synthesis and impact of the branching enzyme
Source: PLoS Comput Biol. 2023 May 19;19(5):e1010694. doi: 10.1371/journal.pcbi.1010694 (PMC10198547; doi:10.1371/journal.pcbi.1010694)
Supplement: S2 Text — Two models for the branching mechanism of GBE are proposed and discussed. (PDF) [file pcbi.1010694.s002.pdf]

# S2: Flexible location *versus* strict location branching models

As briefly introduced in the section The model, paragraph Enzymatic reactions, two assumptions are made for the mechanism of GBE. In the flexible location branching model (used throughout the article) cleaving can equiprobably take place at all positions in the acceptable range, and similarly for branching. Opposite, in the strict location branching model (introduced here for comparison), only the cleaving can take place at various positions, while the branching is fixed to a precise location, i.e. at a given distance from the non-reducing end. For the sake of clarity, these two models and their associated mechanisms are fully detailed in Fig A. Their patterning impact on the CLD is presented in Fig B.

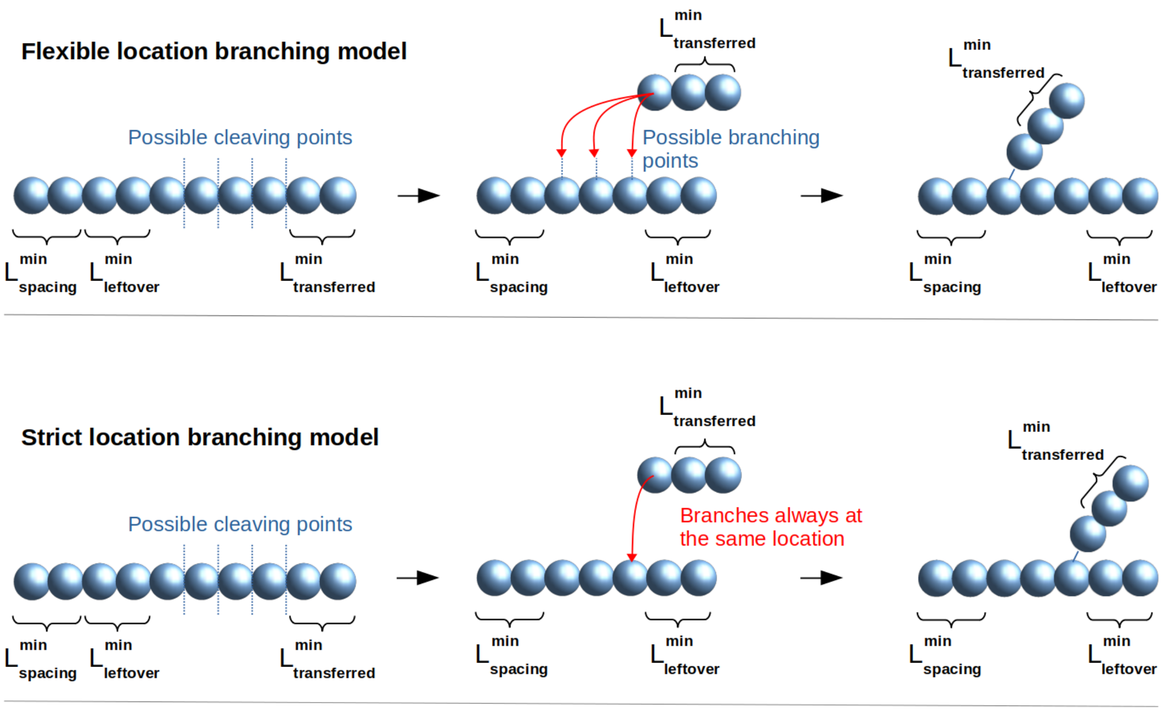


**Fig A. Mechanisms of the flexible location and the strict location branching models.** Both models fulfill the minimal lengths requirement (Eq 1). In the flexible location branching model, the cleaving position is randomly picked from a uniform distribution, and so is that of branching. Instead, in the strict location branching model, both the cleaving and the branching always occur at a specific distance from the non-reducing end.

In Fig B, we compare the two branching models, and vary $\Gamma$ and $\{L^{\text{GBE}}_{\text{spacing}}, L^{\text{GBE}}_{\text{leftover}}, L^{\text{GBE}}_{\text{transferred}}\}$, which results in 12 different plots. From the Elongation to branching ratio section, we learnt that increasing $\Gamma$ spreads the CLD towards higher DPs and reduces the distribution peak typically observed for short chains. In the section Effect of the branching enzyme on the CLD, we observed that increasing the minimal lengths $\{L^{\text{GBE}}_{\text{spacing}}, L^{\text{GBE}}_{\text{leftover}}, L^{\text{GBE}}_{\text{transferred}}\}$ modifies the shape of the CLD, that becomes bi- or

even multi-modal. When decreasing these lengths, the multi-modality is weakened while it is reinforced by a small $\Gamma$.

For $\Gamma = 0.1$, the strict location branching model (red) matches the flexible one (blue). That is not surprising, since in this particular regime, when branching strongly dominates over elongation, branching occurs as soon as possible (DP $> L_{\mathrm{min}}^{\mathrm{GBE}}$), making the two models equivalent.

When $\Gamma$ increases (second and third lines), the two models are not anymore equivalent. In the strict location model, the length of the transferred branch is always equal to $L_{\mathrm{transferred}}^{\mathrm{GBE}}$, while in the flexible location model, longer chains can be transferred. In both cases, the smallest transferable DP is equal to $L_{\mathrm{transferred}}^{\mathrm{GBE}}$ (see section Effect of the branching enzyme on the CLD in the article). For the strict location model the most abundant DP is equal to $L_{\mathrm{transferred}}^{\mathrm{GBE}}$, unlike for the flexible one.

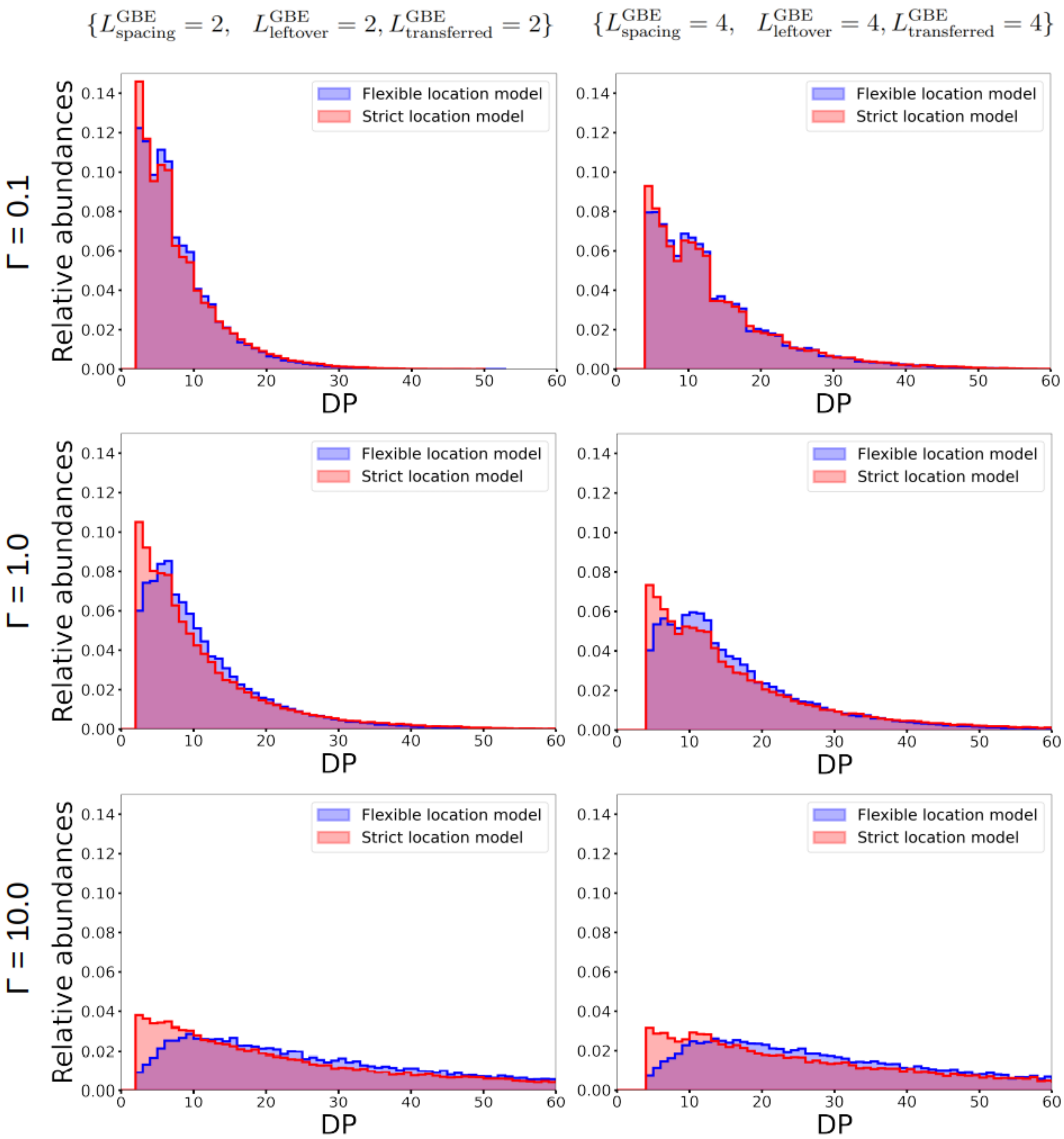


**Fig B. Comparing the impact of the flexible location and the strict location branching models on the CLD.** The CLDs for the flexible location and the strict location branching models are shown in blue and red, respectively. **Top-left:** CLD obtained for small $\Gamma$ (branching regime), with small minimal lengths for GBE. **Top-right:** CLD obtained for small $\Gamma$ (branching regime), with longer minimal lengths for GBE. Increasing the minimal lengths for GBE reinforces the multi-modality. **Middle-left:** CLD obtained for $\Gamma = 1$ (intermediary regime), with small minimal lengths for GBE. Both models loose their multi-modalities. **Middle-right:** CLD obtained for $\Gamma = 1$ (intermediary regime), with increased minimal lengths for GBE. Multi-modality is restored in both models. **Bottom-left:** CLD obtained when increasing $\Gamma$ (elongation regime), with small minimal lengths for GBE. Both conditions contribute to weaken the multi-modality. **Bottom-right:** CLD obtained when increasing both $\Gamma$ and minimal lengths for GBE. The first condition tends to weaken multi-modality while the second one instead reinforces it. As a consequence, multi-modality is observed for the strict location branching model only.
