# Supplementary material for: Stochastic modelling of a three-dimensional glycogen granule synthesis and impact of the branching enzyme
Source: PLoS Comput Biol. 2023 May 19;19(5):e1010694. doi: 10.1371/journal.pcbi.1010694 (PMC10198547; doi:10.1371/journal.pcbi.1010694)
Supplement: S3 Text — The scope of the parameter space searched to find the best set of parameter values is presented. The latter minimises the mean square error between the experimental and the simulated CLDs. Two branching models are compared, resulting in heatmaps with corresponding fitting scores. (PDF) [file pcbi.1010694.s003.pdf]

S3: Scope of the parameter space

We apply our fitting procedure to all possible combinations of $\Gamma \in \{0.1,\ 0.2,\ 0.3,\ 0.4,\ 0.6,\ 0.8,\ 1.0,\ 2.0,\ 5.0\}$, $L^{\mathrm{GBE}}_{\mathrm{spacing}} \in \{1,\ 2,\ 3,\ 4,\ 5,\ 6\}$, $L^{\mathrm{GBE}}_{\mathrm{transferred}} = L^{\mathrm{GBE}}_{\mathrm{leftover}} \in \{2,\ 3,\ 4,\ 5\}$, and $\rho \in \{0\ \mathrm{nm},\ 0.325\ \mathrm{nm},\ 0.650\ \mathrm{nm}\}$, while considering two different branching scenarios, i.e. the flexible location and the strict location branching models. It sums up to a total of 1,296 tested sets of parameter values. The corresponding heat-maps are shown in Fig A for the flexible location and Fig B for the strict location branching models. In both figures, ρ varies across the columns, while $L^{\mathrm{GBE}}_{\mathrm{leftover}} = L^{\mathrm{GBE}}_{\mathrm{transferred}}$ does over the successive rows. On each heatmap, the Y and X axes correspond to $L^{\mathrm{GBE}}_{\mathrm{spacing}}$ and Γ, respectively.

In Fig A, all scores below 20 are highlighted with white squares, and correspond to good fits (arbitrary cut-off chosen as up to twice the best-fit). Noticeably, various sets of parameter values fulfill this criterion and all of them show a small $L^{\mathrm{GBE}}_{\mathrm{spacing}}$ value. As presented in section Comparison to experimental data, paragraph Parameters calibration, the best score is obtained with $\rho = 0.65$ nm (third column). This supports the fact that steric hindrance plays a role in the chain length distribution of real glycogen, although good matches also exist with $\rho = 0$ nm, in which the CLD is not impacted by steric hindrance. When focussing on the good scores (dark blue cells), it appears that changing the elongation to branching ratio Γ can be compensated by varying $L^{\mathrm{GBE}}_{\mathrm{spacing}}$, or the two other minimal lengths.

Opposite, when considering parameter values that are typically reported in the literature, we systematically obtain very poor (i.e. high) scores, even upon varying Γ. This case is highlighted in red and discussed in the section Comparison to experimental data, paragraph Parameters calibration. In general terms, for each heatmap, we observe that $L^{\mathrm{GBE}}_{\mathrm{spacing}} \geq 4$ corresponds to poor scores, i.e. $\mathcal{S} \geq 30$. This effect is even more pronounced if $L^{\mathrm{GBE}}_{\mathrm{spacing}} \geq 6$, i.e. $\mathcal{S} \geq 66.3$. Thus, with these sets of parameter values, our model is not able to reproduce the experimental CLD data by Sullivan and coworkers used for fitting throughout this article [1]. Regarding the enzyme mechanism, this suggests that GBE is able to branch much closer than 4 glucose units away from an existing sister chain. Similarly, if $L^{\mathrm{GBE}}_{\mathrm{transferred}} = L^{\mathrm{GBE}}_{\mathrm{leftover}} \geq 3$, we are not able to fit the experimental data by Sullivan and coworkers. Therefore, these minimal lengths must be shorter than those typically reported in the literature.

In Fig B, we present analogous results for the strict location model. Overall, the fitting procedure returns much poorer scores than for the flexible location branching model (see Fig A). This is due to the fact that strict branching locations almost systematically lead to a multi-modal distribution, which is not the case of the experimental CLD data set fitted here [1]. Although multi-modality can be cleared by increasing Γ, this would lead to a reduction of the distribution peak and a shift of the overall distribution towards higher DPs. For the experimental data considered, we could not find a good trade-off, corresponding to good fitting scores. As a result, the strict location branching model seems to be very unlikely, that is why we instead choose the flexible location branching model for this study.

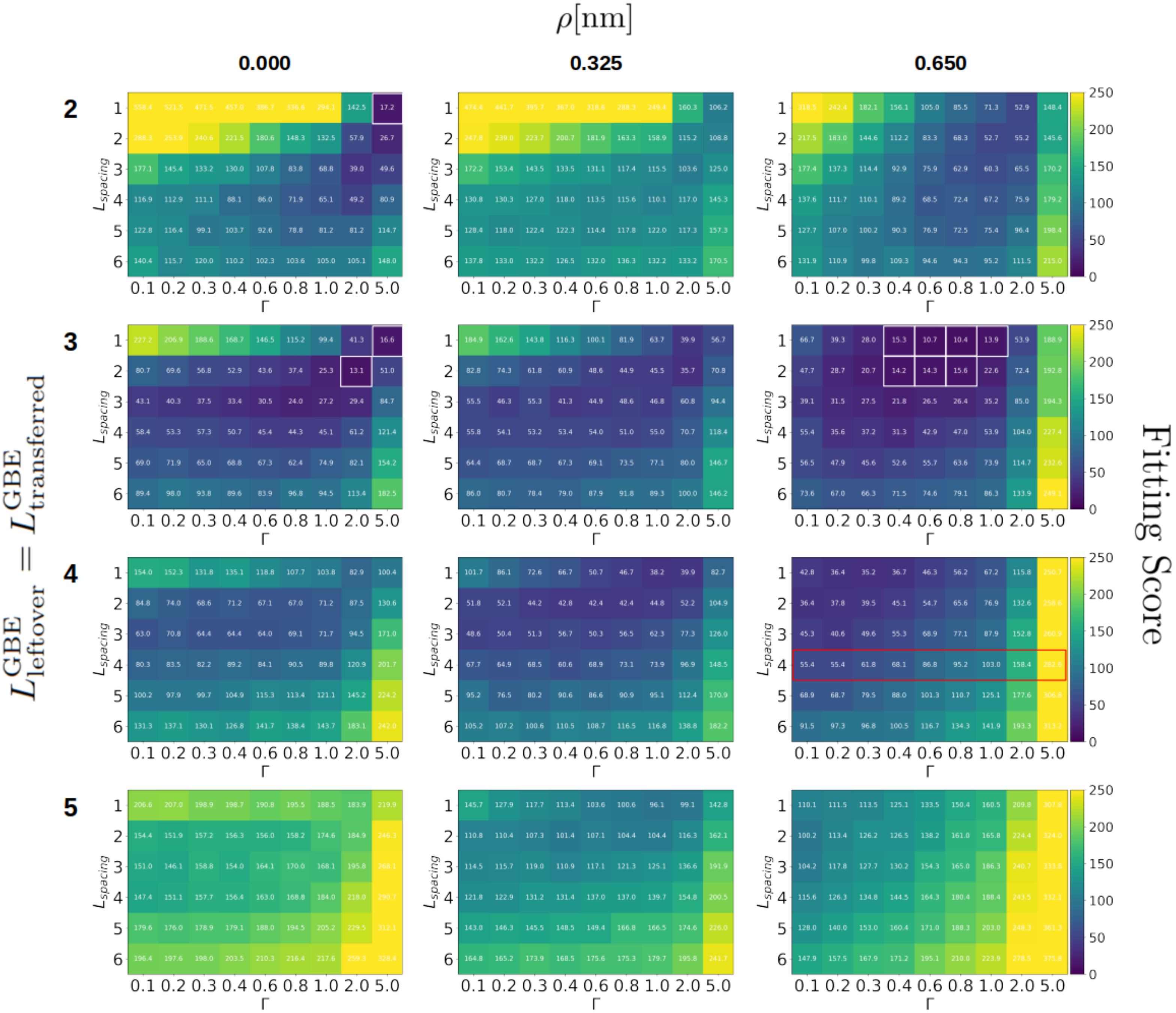

Fig A. Heat-maps showing fitting scores for the flexible location branching model. The three columns show 3 different radii for the glucose units ($\rho = 0$ nm, 0.325 nm, and 0.650 nm). The 4 rows correspond to distinct values of $L_{\text{transferred}}^{\text{GBE}}$ (2, 3, 4, and 5) with $L_{\text{leftover}}^{\text{GBE}} = L_{\text{transferred}}^{\text{GBE}}$. For each heat-map, the values for $L_{\text{spacing}}^{\text{GBE}}$ are on the Y axis, while the Γ ratio is on the X axis. Each cell is characterised by a unique set of parameter values for $\{\rho, \Gamma, L_{\text{spacing}}^{\text{GBE}}, L_{\text{leftover}}^{\text{GBE}}, L_{\text{transferred}}^{\text{GBE}}\}$. White squares show good scores ($\mathcal{S} \leq 20$), while the red rectangle ($\mathcal{S} \geq 55.4$) shows the scores obtained for values of GBE's minimal lengths as typically assumed in the literature.

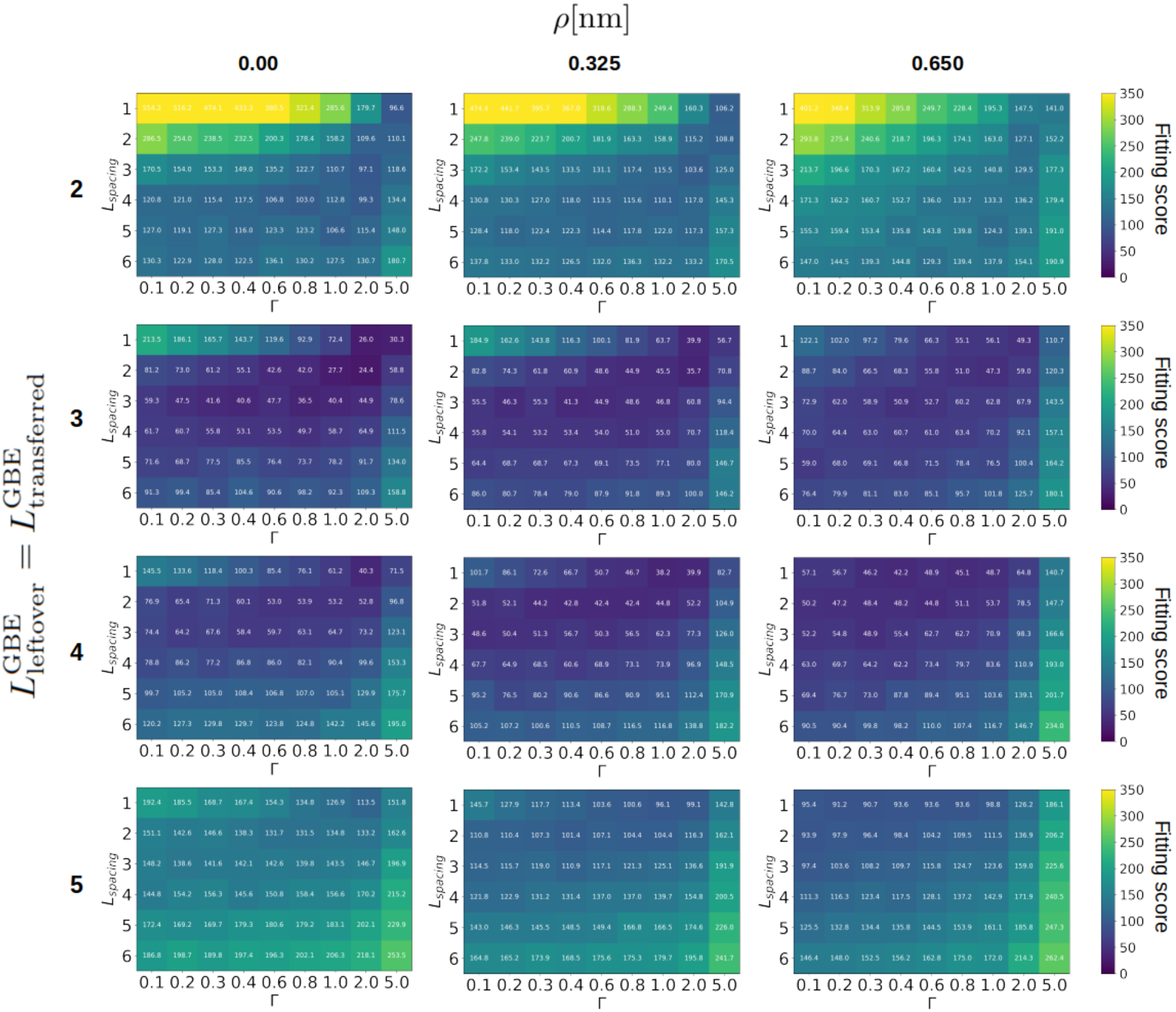

Fig B. Heat-maps showing fitting scores for the strict location branching model. The three columns show 3 different radii for the glucose units ($\rho = 0$ nm, 0.325 nm, and 0.650 nm). The 4 rows correspond to distinct values of $L^{\mathrm{GBE}}_{\mathrm{transferred}}$ (2, 3, 4, and 5) with $L^{\mathrm{GBE}}_{\mathrm{leftover}} = L^{\mathrm{GBE}}_{\mathrm{transferred}}$. For each heat-map, the values for $L^{\mathrm{GBE}}_{\mathrm{spacing}}$ are on the Y axis, while the Γ ratio is on the X axis. Each cell is characterised by a unique set of parameter values for $\{\rho, \Gamma, L^{\mathrm{GBE}}_{\mathrm{spacing}}, L^{\mathrm{GBE}}_{\mathrm{leftover}}, L^{\mathrm{GBE}}_{\mathrm{transferred}}\}$. Overall, the scores are much higher than those for the flexible location branching model (see Fig A), indicating poorer fits. Specifically, no scores below the threshold $\mathcal{S} = 20$ are found.

References

1. Sullivan MA, Nitschke S, Skwara EP, Wang P, Zhao X, Pan XS, et al. Skeletal Muscle Glycogen Chain Length Correlates with Insolubility in Mouse Models of Polyglucosan-Associated Neurodegenerative Diseases. Cell Reports. 2019;27(5):1334–1344.e6. doi:https://doi.org/10.1016/j.celrep.2019.04.017.
