# Supplementary material for: Stochastic modelling of a three-dimensional glycogen granule synthesis and impact of the branching enzyme
Source: PLoS Comput Biol. 2023 May 19;19(5):e1010694. doi: 10.1371/journal.pcbi.1010694 (PMC10198547; doi:10.1371/journal.pcbi.1010694)
Supplement: S4 Text — Discussion about the expected value for the A:B ratio in a purely probabilistic approach. (PDF) [file pcbi.1010694.s004.pdf]

S4: Probabilistic approach to the A:B ratio

In this section, we describe why the A:B ratio must be equal to 1 when no biological nor biophysical properties of the system are considered, but pure probabilities. As presented in section Glycogen structure using the fitted parameters in the main article, we note as two distinct reactions, branching on either an A or a B chain:

$$R_{A\to AB}: \quad \mathrm{A} \xrightarrow{\mathrm{GBE}} \mathrm{B} + \mathrm{A}_{\mathrm{new}}$$
$$R_{B\to AB}: \quad \mathrm{B} \xrightarrow{\mathrm{GBE}} \mathrm{B} + \mathrm{A}_{\mathrm{new}}\ .$$

In this purely mathematical framework, A and B chains have the same chance to react, so that the probability for a reaction to occur only depends on the respective number of A (noted N_A) and B (noted N_B) chains. We sketch the associated probability tree in Fig A. The tree is symmetric, and the number of paths to a given state follows the binomial coefficients. The horizontal dotted line corresponds to $N_A - N_B = 0$, when there are as many A as B chains. We know that in the example of flipping a coin i times, the central limit theorem tells us that the distribution of the difference in the number of heads and tails, tends to a normal distribution centered in 0 when i tends to infinity. This case corresponds to heads and tails having equal probabilities. In our case, a given state of the probability tree (N_A, N_B) leads either to the state (N_A, N_B+1) with probability $p(R_{A\to AB}) = \frac{N_A}{N_A+N_B}$, or to (N_A+1, N_B) with probability $p(R_{B\to AB}) = \frac{N_B}{N_A+N_B}$. It means that the probability to go from one state to the next one depends on the state of the system, in a way that the probability to come closer to $N_A - N_B = 0$ (horizontal dotted line) is always higher than that of spreading away. Additionally, the distribution of the probabilities remains symmetric with respect to the case $N_A = N_B$. Based on these considerations, the mean value $\overline{N_A - N_B} = 0$ and the distribution is even more peaked than in the simple case of flipping a coin. Since the mean value $\overline{N_A - N_B} = 0$, the mean ratio $\overline{\frac{N_A}{N_B}} = 1$, in other words the A:B ratio is 1.

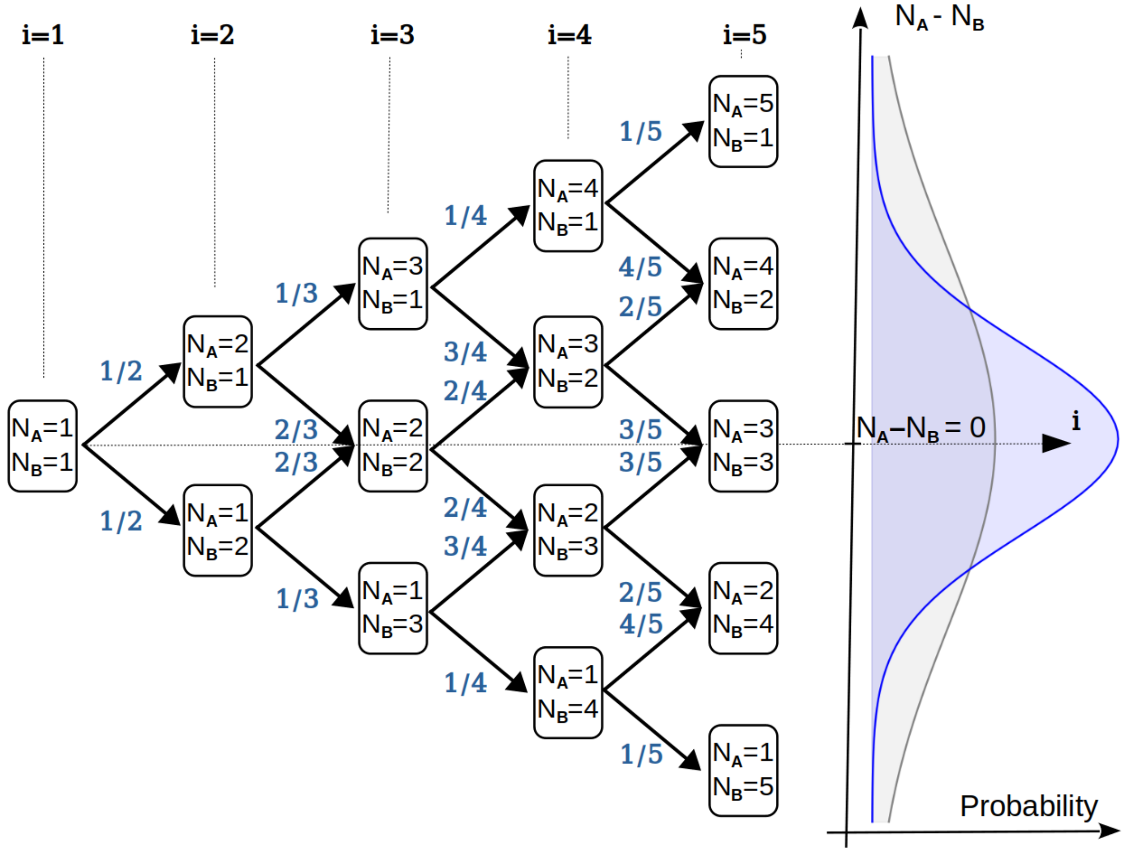

Fig A. Probability tree of forming a given number of chains of type A (N_A) and B (N_B). Directions that spread the tree are unfavoured, while those oriented towards the center of the tree (reducing the absolute value $|N_A - N_B|$) are favoured, proportionally to the difference $|N_A - N_B|$. Therefore, for high numbers of branching reactions (noted i) the distribution (blue) is centered around $N_A - N_B = 0$, and is thinner than a binomial distribution (grey).
