# Supplementary material for: Stochastic modelling of a three-dimensional glycogen granule synthesis and impact of the branching enzyme
Source: PLoS Comput Biol. 2023 May 19;19(5):e1010694. doi: 10.1371/journal.pcbi.1010694 (PMC10198547; doi:10.1371/journal.pcbi.1010694)
Supplement: S5 Text — Explanation of the Gillespie direct method used to simulate the glycogen synthesis reactions. Flow diagram showing the main steps of the simulations. (PDF) [file pcbi.1010694.s005.pdf]

S5: Numerical procedures

A brief reminder of the Gillespie "direct" method

In this subsection, we summarise the main steps of the so-called "direct" Gillespie method that has been introduced by Daniel Gillespie in 1976 [1] and further popularised in 1977 [2]. It consists in splitting the probability:

$$\begin{aligned}\mathcal{P}(\mu,\tau) = &\text{ Probability for the next reaction to be the reaction } \mathcal{R}_\mu \\ &\text{ and to occur between time } \tau \text{ and } \tau + d\tau,\end{aligned}$$

into two terms, using conditional probability. $\mathcal{P}(\mu,\tau)$ can be written as follows:

$$\mathcal{P}(\mu,\tau) = \mathcal{P}(\tau)\cdot\mathcal{P}(\mu|\tau) \tag{1}$$

where $\mathcal{P}(\tau)$ is the probability that no reaction occurs between $t = 0$ and $t = \tau$, and $\mathcal{P}(\mu|\tau)$ is the probability that, given that a reaction occurs during the infinitesimal time τ and $\tau + d\tau$, it is the reaction $\mathcal{R}_\mu$. Gillespie showed that:

$$\mathcal{P}(\tau) = a\cdot\exp(-a\tau), \tag{2}$$

where a is the sum of all propensities a_μ, and $a_\mu \cdot dt$ is the probability for the reaction μ to occur in the next infinitesimal time interval dt. Additionally,

$$\mathcal{P}(\mu|\tau) = \frac{a_\mu}{a}. \tag{3}$$

The idea underpinning the algorithm is to draw random numbers that determine the next reaction and reaction time, according to these probabilities. (Eq 2) can be integrated to get the probability distribution function $F(t)$ from the probability density function $\mathcal{P}(t)$.

$$F(t) = \int_0^t \mathcal{P}(t')\cdot dt', \tag{4}$$

such that any $F(t_0)$ is the probability that t is less than t_0. Using the inversion generating method described in details in the Appendix of [2], Gillespie showed that the time τ can be generated using a pseudo-random number r_1 following a uniform distribution in $[0,1]$, such that:

$$\tau = \frac{1}{a}\cdot\ln(1/r_1). \tag{5}$$

To determine which reaction takes place at time τ, one draws a second pseudo-random number r_2 following a uniform distribution in $[0,1]$, and looks in which of the following intervals it falls. For N possible reactions, the reaction $\mu \in (1,..,N)$ will be selected if:

$$\sum_{j=1}^{\mu}\frac{a_j}{a} \le r_2 < \sum_{j=1}^{\mu+1}\frac{a_j}{a}. \tag{6}$$

The couple $\{\mu,\tau\}$ determines the reaction μ and the increment of time $t \leftarrow t + \tau$ to be implemented. The propensities of the distinct reactions of the system are computed based on rate laws. As a first assumption, in our model, we use mass-action kinetics with rate constants $k = 1$. Therefore, the respective propensities for the elongating enzyme (a_{GS}) and the branching enzyme (a_{GBE}) write:

$$a_{\text{GS}} = c_{\text{GS}} \cdot S_{\text{GS}}$$
$$a_{\text{GBE}} = c_{\text{GBE}} \cdot S_{\text{GBE}},$$

where c_{GS} and c_{GBE} are parameters that can force the system into either elongation or branching, depending on the previously introduced ratio $\Gamma = \frac{c_{\text{GS}}}{c_{\text{GBE}}}$. Besides, S_{GS} and S_{GBE} are the number of available substrate chains for GS and GBE, respectively. It is important to recall that we are interested in the phenomenology of the system. In this context, simply assuming mass-action kinetics is sufficient to investigate the different elongation and branching regimes of interest.

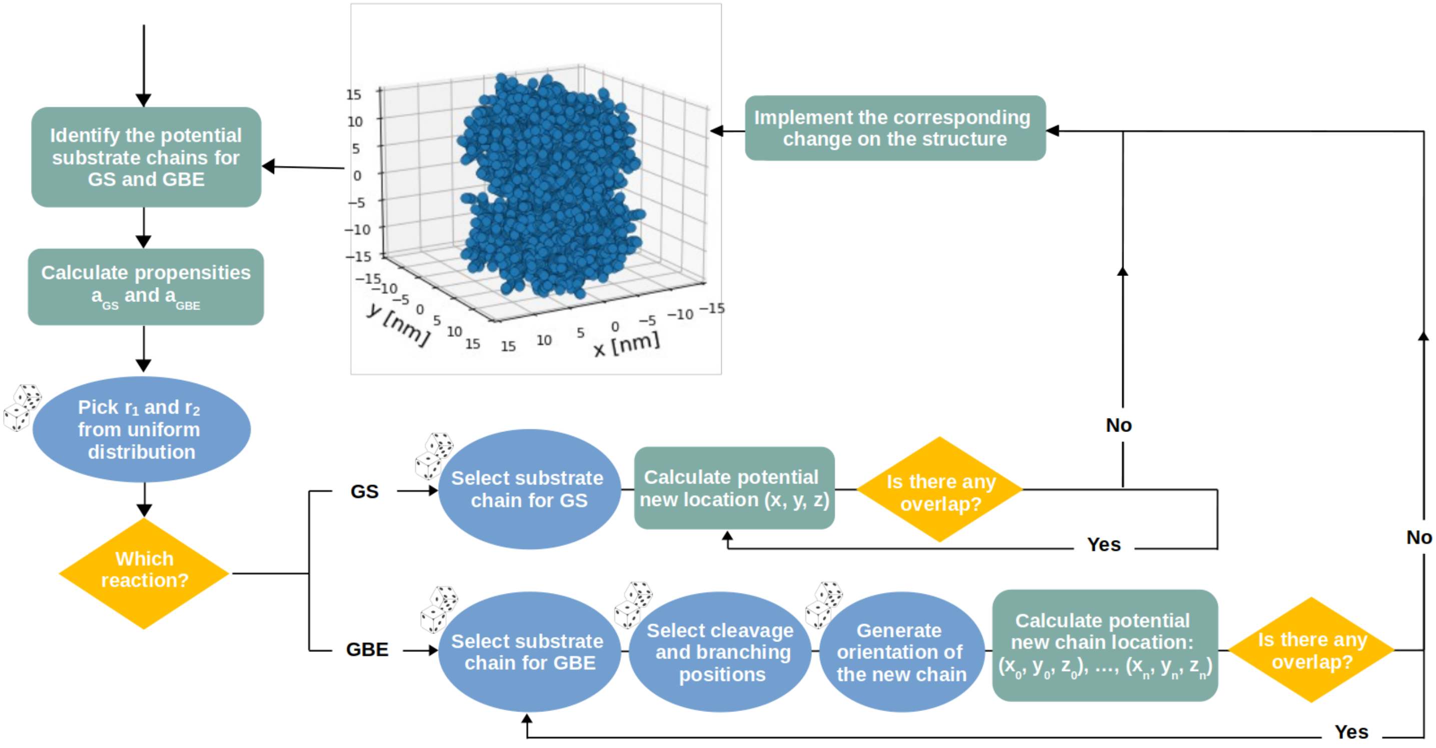

Fig A. Algorithm flow diagram. This scheme shows the main steps of our algorithm. The green round rectangles denote deterministic calculations or actions. The blue ovals involve stochastic steps relying on random numbers. Finally, the yellow diamonds are conditional decisions (or "if" statements).

Overall simulation algorithm

The main steps of our algorithm are outlined in Fig A . The overall loop, in which the biochemical reactions are simulated, begins by analysing the granule structure and identifying the available substrate chains for GS and GBE. Based on this information, the propensity for each reaction is calculated, using a mass-action kinetic approach as described above. The Gillespie direct method randomly determines the next reaction and its duration. If the reaction is an elongation, the code selects one of the potential substrate chains at random, and analyses the granule structure to check for overlaps. If there is none,

the glucose monomer is added to the substrate chain. Otherwise, another substrate chain is selected at random, and the overlap criterion is checked again. If the reaction is a branching, the code proceeds in the same way, but it verifies that there is no overlap for the entire new daughter chain.

References

1. Gillespie DT. A general method for numerically simulating the stochastic time evolution of coupled chemical reactions. Journal of Computational Physics. 1976;22(4):403–434. doi:https://doi.org/10.1016/0021-9991(76)90041-3.

2. Gillespie DT. Exact stochastic simulation of coupled chemical reactions. The Journal of Physical Chemistry. 1977;81(25):2340–2361. doi:10.1021/j100540a008.
